# Thrombin generation potential in the presence of concizumab and rFVIIa, APCC, rFVIII, or rFIX: *In vitro* and *ex vivo* analyses

**DOI:** 10.1111/jth.15323

**Published:** 2021-05-06

**Authors:** Marianne Kjalke, Mads Kjelgaard‐Hansen, Søren Andersen, Ida Hilden

**Affiliations:** ^1^ Global Drug Discovery Novo Nordisk A/S Måløv Denmark; ^2^ Global Development Novo Nordisk A/S Bagsvaerd Denmark

**Keywords:** FEIBA, hemophilia A, hemophilia B, recombinant factor VIII, recombinant factor VIIa

## Abstract

**Background:**

The anti‐tissue factor plasma inhibitor monoclonal antibody concizumab is under clinical investigation for subcutaneous prophylaxis of hemophilia A/B (HA/HB) with or without inhibitors. Breakthrough bleeds while on concizumab prophylaxis may be treated with bypassing agents (recombinant activated factor VIIa [rFVIIa] and activated prothrombin complex concentrate [APCC]), or with factor VIII (FVIII) or factor IX (FIX).

**Objectives:**

To evaluate the effect of combining concizumab with rFVIIa, APCC, rFVIII, and rFIX on thrombin generation (TG) potential.

**Methods:**

Pooled HA plasma was spiked *in vitro* with concizumab alone or together with rFVIIa, APCC, or rFVIII. rFVIIa, APCC, and rFVIII were added *ex vivo* to plasma from HA patients receiving concizumab prophylaxis. Pooled HB plasma was spiked with concizumab alone or together with rFIX. TG potential was measured after initiation with tissue factor.

**Results:**

Concizumab increased thrombin peak in a concentration‐dependent manner. Adding rFVIIa, APCC, rFVIII, or rFIX caused a further increase in thrombin peak. The effects of concizumab and rFVIIa, APCC, rFVIII, or rFIX were mainly additive, with no or up to maximally ~25% extra effect caused by drug‐‐drug interaction. No strong synergistic effects were observed upon combining concizumab with rFVIIa, APCC, rFVIII, or rFIX. The thrombin peak obtained with 0.5 IU/ml rFVIII or rFIX in the presence of concizumab was on occasion slightly higher, but mostly comparable to the thrombin peak with 1 IU/ml rFVIII or rFIX in the absence of concizumab.

**Conclusion:**

rFVIIa, APCC, rFVIII, and rFIX enhanced plasma TG potential in the presence of concizumab. Dose levels of concomitant use should be adjusted accordingly to balance potential safety concerns while maintaining the necessary hemostatic effect. Please see the video in the Supplementary Material for an animated summary of the data presented.

**Essentials**

- Recombinant activated factor VIIa (rFVIIa), activated prothrombin complex concentrate (APCC), factor VIII (FVIII), or factor IX (FIX) may be used to treat bleeds in patients on concizumab prophylaxis.
- Thrombin generation potential with concizumab−rFVIIa/APCC/rFVIII/rFIX combination was analyzed.
- The effects of combining concizumab with rFVIIa/APCC/rFVIII or rFIX were mainly additive.
- Data support rFVIIa/APCC/rFVIII or rFIX use for breakthrough bleeds with concizumab prophylaxis.

​

​

## 1 INTRODUCTION

Hemophilia is characterized by an increased bleeding tendency, manifesting as spontaneous bleeding that affects the joints, muscles, and soft tissues, and prolonged bleeding following trauma or surgery.^1^ The underlying cause of the disease is the absence or deficiency of functional coagulation factor VIII (FVIII; hemophilia A [HA]) or factor IX (FIX; hemophilia B [HB]),^1^ which in turn leads to reduced thrombin generation (TG) potential. In order to prevent bleeding episodes and related complications, HA and HB patients receive prophylactic treatment with FVIII or FIX, respectively.^2^ Patients with hemophilia and inhibitors are commonly treated with bypassing agents, such as recombinant activated factor VII (rFVIIa; NovoSeven^®^) or activated prothrombin complex concentrate (APCC; FEIBA^®^).^3^ However, outcomes with bypassing agents are variable and tend to be inferior to factor replacement therapy.^4^, ^5^ These limitations have prompted a shift in focus toward the development of novel, non‐replacement therapies for patients with hemophilia.

One such non‐replacement hemostatic agent is concizumab, a humanized monoclonal antibody directed against the Kunitz 2 domain of tissue factor pathway inhibitor (TFPI), currently in phase 3 clinical development as a subcutaneous prophylactic treatment for HA and HB, both with and without inhibitors.^6^, ^7^ TFPI is the principal regulator of the initiation phase of coagulation.^8^, ^9^, ^10^ The initiation phase results from injury to the vessel wall and exposure of tissue factor (TF) on subendothelial cells to the blood, allowing binding of FVIIa to TF. The complex between TF and FVIIa activates FIX to FIXa and factor X (FX) to FXa. This initial FVIIa/TF‐mediated FXa generation is regulated by TFPI, which binds to the active site of FXa. As soon as TFPI is complexed with FXa, TFPI/FXa binds FVIIa/TF, thereby downregulating initiation of coagulation. In addition, TFPI inhibits early forms of the prothrombinase complex (activated factor V [FVa]/FXa) via interaction with the B‐domain of partially activated FVa, that is, platelet FVa and FXa‐activated FVa.^10^ After downregulation of the initiation phase, FX is converted to FXa through the activity of the FVIIIa/FIXa complex on the activated platelet, thereby generating a thrombin burst.^9^, ^11^ In hemophilia, the absence of FVIII or FIX results in impaired TG potential on the activated platelet. By minimizing the inhibitory activity of TFPI, concizumab enhances the initiation phase of coagulation, leading to the production of sufficient quantities of FXa and subsequently thrombin to achieve hemostasis in the absence of FVIII or FIX. As concizumab acts independently of the presence or absence of FVIII or FIX, it is suitable for the treatment of patients both with HA and HB, regardless of their inhibitor status.^6^, ^7^

In phase 2 trials, once‐daily prophylaxis with concizumab was shown to be efficacious and well tolerated both in hemophilia patients with and without inhibitors (explorer4 [NCT03196284] and explorer5 [NCT03196297] trials, respectively).^7^ The subsequent phase 3 trials, explorer7 (NCT04083781) and explorer8 (NCT04082429), as well as the extension phase of explorer5, were paused by Novo Nordisk and were subsequently temporarily placed on clinical hold by the Food and Drug Administration in March 2020 due to reports of non‐fatal, thrombotic events (TEs) in three patients. All three patients had thrombotic risk factors at baseline and had used concomitant hemostatic medication (rFVIIa or FVIII) on the day of, and, in two cases, in the days before the TE.^12^ Novo Nordisk developed a risk mitigation plan based on in‐depth, cross‐functional analysis of available data from phase 2 and 3 clinical trials with concizumab, including data from the three patients who experienced TEs, which led to the clinical hold being lifted in August 2020 and the phase 3 trials resuming.^13^

The aim of the study presented herein was to evaluate the *in vitro* effect of the combination of concizumab and the bypassing agents rFVIIa and APCC, as well as rFVIII and rFIX, on TG potential. To this end, the effect of adding rFVIIa, APCC, or rFVIII to a HA plasma pool spiked with concizumab and to samples from HA patients having received concizumab prophylaxis in a clinical trial setting was assessed. Furthermore, we performed an additional analysis to identify the effect that spiking with rFIX had on HB plasma pool samples in the presence or absence of concizumab.

## 2 MATERIALS AND METHODS

### 2.1 *In vitro*/*ex vivo* spiking experiments

#### 2.1.1 Spiking of a HA plasma pool with rFVIIa, APCC, or rFVIII in the absence or presence of concizumab

Concizumab (Novo Nordisk) was added to a HA plasma pool (George King Bio‐Medical Inc.) either alone or in combination with rFVIIa (NovoSeven^®^, Novo Nordisk), APCC (FEIBA^®^, Takeda) or rFVIII (NovoEight^®^, Novo Nordisk).

#### 2.1.2 Spiking of plasma samples from HA patients with and without inhibitors on concizumab prophylaxis (explorer4 and explorer5 trials) with rFVIIa or APCC

*Ex vivo* plasma samples from 15 patients with HA and inhibitors were obtained from the explorer4 trial.^7^ Samples from patients with HA without inhibitors were obtained from the explorer5 trial (*n* = 30) and were used to adjust the spiking concentrations of APCC in subsequent analyses of explorer4 samples. Plasma samples were collected at baseline, that is, before the first concizumab administration (visit 1), as well as after steady‐state concizumab concentrations had been reached (visits 5, 6, and 7 after 8, 12, and 16 weeks of concizumab treatment, respectively). Concizumab levels in plasma were determined by ELISA.^7^ Either rFVIIa or APCC were added to baseline and steady‐state plasma samples. In addition, human rFVIII was added to plasma pool and baseline (visit 1) explorer5 samples and porcine rFVIII (Obizur^®^, Takeda) was added to baseline (visit 1) explorer4 samples in order to establish a reference of TG potential in the presence of normal FVIII level (see Results and Figure S1).

#### 2.1.3 The effect of adding rFVIII to plasma samples from HA patients without inhibitors (explorer5)

rFVIII was added to a total of 33 plasma samples from eight patients with HA without inhibitors receiving prophylaxis with concizumab with levels of free TFPI, that is, TFPI not in complex with concizumab, below the lower limit of quantification of the assay (9.6 ng/ml) and pre‐dose samples available for seven of these patients.

#### 2.1.4 Spiking of a HB plasma pool with recombinant FIX (rFIX) in the absence or presence of concizumab

rFIX (Benefix^®^, Pfizer) was added to a HB plasma pool (George King Bio‐Medical Inc.) in the absence or presence of concizumab.

### 2.2 Thrombin generation assay

The effect of concizumab and rFVIIa, APCC, rFVIII, or rFIX was measured in a TG assay (Thrombinoscope™) using PPP‐Reagent LOW containing 1 pM TF and phospholipids.^14^ The effect of rFVIIa, APCC, rFVIII, and rFIX in the absence and presence of concizumab was compared by thrombin peak data, as all of these hemostatic agents influenced the thrombin peak. rFVIIa and APCC resulted in a shorter lag time, while concizumab, rFVIII, and rFIX had no or modest effects on lag time, rendering this parameter suboptimal for evaluation of combined effects with concizumab. Values obtained for endogenous thrombin potential (ETP) were qualitatively similar to the values for thrombin peak.

### 2.3 Statistical analysis

#### 2.3.1 Analysis of pooled hemophilia plasma

For HA/HB plasma pool data, a three‐way analysis of variance (ANOVA) was applied based on the following factors: experiment number (from a total of three repeats each), concentration of bypassing agent/rFVIII (none; rFVIIa 25 or 75 nM; APCC 0.5 or APCC 1 U/ml; rFVIII 0.25, 0.5, or 1 IU/ml), and concizumab concentration (0, 150, 450, or 1500 ng/ml initially or 1500, 4500, and 15,000 ng/ml for a subsequent analysis and in combination with rFVIII). The same principle was applied to analyze the effect of rFIX (0.25, 0.5, or 1 IU/ml) in a HB plasma pool. Interactions among the spiking factors, drug concentration, and concizumab concentration were included in the model.

The effect of either bypassing agent (rFVIIa or APCC) on the background of each concizumab concentration was defined as the difference to the drug‐concentration level “none.” These effects were estimated from the three‐way ANOVA model. To investigate the differences in drug‐drug interactions from the ANOVA model, the effects of the different concentrations of rFVIIa and APCC on the background of the different concizumab concentrations were compared to the effect they had on a background without concizumab.

#### 2.3.2 Analysis of explorer4 and explorer5 samples spiked with rFVIIa or APCC

Samples taken at visits 5, 6, and 7 (after 8, 12, and 16 weeks of concizumab treatment, respectively) were considered representative of the behavior of concizumab at steady state. The effect of a bypassing agent on the concizumab‐free background was defined as the difference to no spiking with bypassing agents at visit 1 (before the start of concizumab prophylaxis). The effect of a bypassing agent on the concizumab background was defined as the difference to no spiking with bypassing agents at visit 5, 6, and 7. In order to establish drug‐drug interactions, the effects of either bypassing agent at the indicated concentrations on the background of concizumab versus no concizumab were compared. Details on the statistical model applied are available in the supporting information.

#### 2.3.3 Analysis of explorer5 samples spiked with rFVIII

Peak heights for each patient were averaged for each level of rFVIII, resulting in a 2 x 3, two‐way table: concizumab (no, yes) × rFVIII (0.5; 1 and 1.5 IU/ml). A three‐way ANOVA was applied to analyze these summarized data, with the three factors being patient, concizumab leveli, and rFVIII level.

## 3 RESULTS

### 3.1 The effect of combining concizumab and rFVIIa

#### 3.1.1 Spiking of a HA plasma pool with rFVIIa and concizumab

**FIGURE 1:**
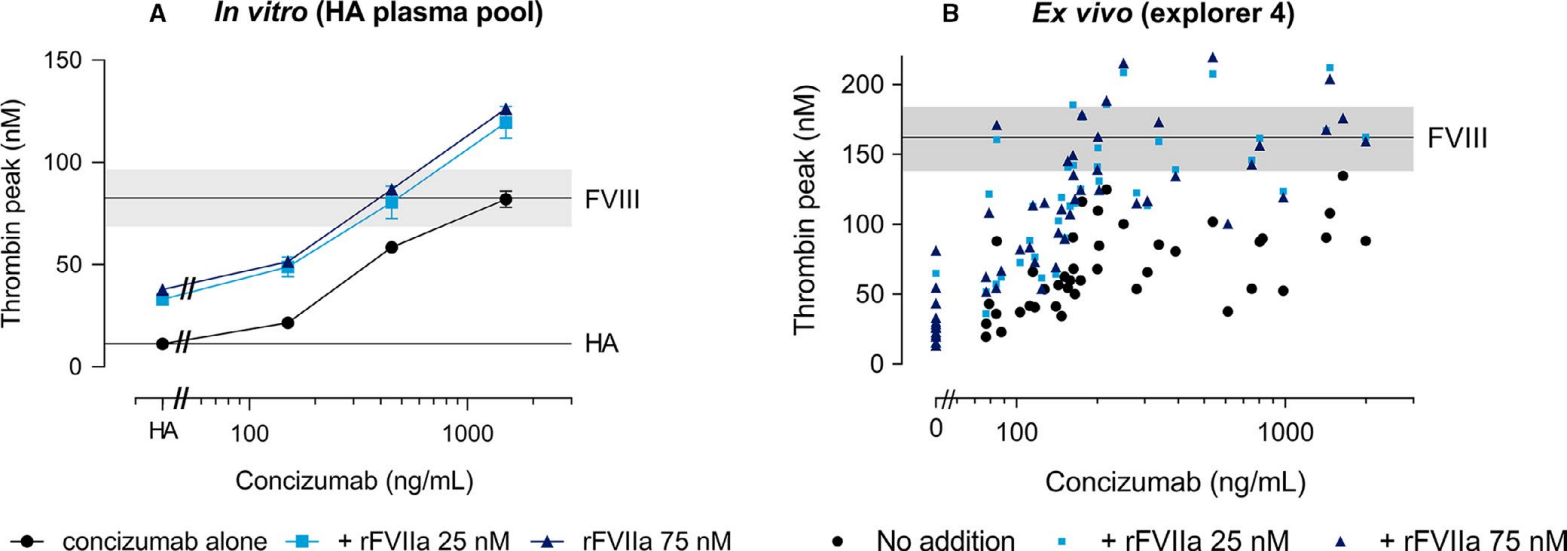
The effect of activated recombinant factor VII (rFVIIa) on thrombin generation (TG) in the absence and presence of concizumab when added *in vitro* to a hemophilia A (HA) plasma pool (A) and *ex vivo* in plasma samples from HA patients with inhibitorsdosed concizumab (B). Concizumab alone or in combination with rFVIIa at 25 or 75 nM was added to a HA plasma pool (A). rFVIIa (25 or 75 nM) was added to plasma samples from HA patients with inhibitors participating in the explorer4 trial at baseline before concizumab administration (data points at 0 ng/ml concizumab) and after 8, 12, and 16 weeks of daily subcutaneous concizumab administration (B). TG was measured after initiation of the assay with 1 pM tissue factor and values for the thrombin peak are depicted (A: mean ± 1 standard deviation [SD] of *n* = 3; B: single measurements of all plasma samples). Values shown in (B) are baseline‐subtracted, that is, values for pre‐concizumab samples without spiking are subtracted. The upper black horizontal line and the gray area depicts (A) the mean ± 1 SD of the thrombin peak observed upon addition of recombinant factor VIII (rFVIII) at 1 IU/ml to the HA plasma pool to reflect normal FVIII plasma level, or (B) the mean and 95% confidence interval (CI) of rFVIII (1 IU/ml) added to non‐inhibitor HA plasma (explorer5 baseline samples). The bottom horizontal line (A) represents the mean ± 1 SD of the thrombin peaks observed for the HA plasma pool without spiking

rFVIIa and concizumab were added to a HA plasma pool alone or in combination, and TG potential was measured. rFVIIa concentrations of 25 and 75 nM, slightly above the plasma level obtained after administration of the recommended approved doses of 90 and 270 µg/kg, respectively,^15^ were applied. rFVIIa at 25 or 75 nM increased the thrombin peak both in the absence and presence of concizumab (*P* < .0001, Figure 1A). There was no apparent difference in the assay between 25 and 75 nM rFVIIa, reflecting that TG assay in platelet‐poor plasma is not sensitive to these differences in rFVIIa concentrations. The combination of concizumab and rFVIIa resulted in thrombin peaks within or slightly above the levels observed when adding rFVIII to normal FVIII plasma levels (1 IU/ml; Figure 1A).

The effects of concizumab and rFVIIa were primarily additive, with the additive effects accounting for all of the observed effects on the thrombin peak when combining rFVIIa (25 or 75 nM) with 150–450 ng/ml concizumab, and for 85.5% and 84.7% of the observed effects upon combining rFVIIa 25 nM and 75 nM, respectively, with 1500 ng/ml concizumab (Figure S2). Overall, a statistically significant drug‐drug interaction was only seen at a concizumab concentration of 1500 ng/ml for both rFVIIa at 25 and 75 nM (Figure S2).

Increased concentrations of concizumab at 4500 and 15,000 ng/ml, reflecting the maximal levels observed in the clinical setting, led to only a modest or no further increase in thrombin peak compared to 1500 ng/ml concizumab, independent of the absence or presence of rFVIIa (Figure S3A). This suggests that plasma TFPI is saturated or close to saturation at 1500 ng/ml concizumab.

#### 3.1.2 The effect of spiking patient plasma samples (explorer4) with rFVIIa

The addition of rFVIIa to plasma samples from 15 patients with inhibitor‐complicated HA receiving concizumab prophylaxis (explorer4 trial) led to an increase in thrombin peaks in both baseline samples taken before concizumab treatment (visit 1; Figure 1B [0 ng/ml data point for concizumab]) and steady‐state samples taken while patients were receiving concizumab (visits 5–7, after 8–16 weeks of treatment, respectively). Examples from three patients, illustrating donor‐to‐donor assay response variation, are provided in Figure S4. Spiking with rFVIIa increased the thrombin peak at all concizumab concentrations (Figure 1B). For most concizumab‐containing samples, the thrombin peak after adding rFVIIa at 25 or 75 nM was within or below the thrombin peak range established for adding a normal level of rFVIII (1 IU/ml) to non‐inhibitor (explorer5) samples. In a few samples, the maximal increase in thrombin peak was modestly above the upper 95% confidence interval (CI) of the range obtained for a normal level of FVIII (Figure 1B).

The isolated effects of concizumab and rFVIIa were compared to the observed effects in samples in which both concizumab and rFVIIa were present in order to assess drug‐drug interaction. The effect of rFVIIa and concizumab were mainly additive; however, statistically significant increased thrombin peak was observed in samples containing both concizumab and 75 nM rFVIIa, demonstrating the presence of drug‐drug interaction (i.e., a synergistic effect) between concizumab and rFVIIa (Figure S5). Due to limited amount of plasma, spiking with 25 nM rFVIIa was not performed with all plasma samples and evaluation of drug‐drug interaction between concizumab and this concentration of rFVIIa was therefore not performed. The additive effects of concizumab and 75 nM rFVIIa accounted for 78 ± 14% of the observed effects on the thrombin peak in samples containing both concizumab and rFVIIa, while the remaining effect can be attributed to drug‐drug interactions (Figure S6).

### 3.2 The effect of combining concizumab and APCC

#### 3.2.1 Spiking of HA plasma pool with APCC and concizumab

**FIGURE 2:**
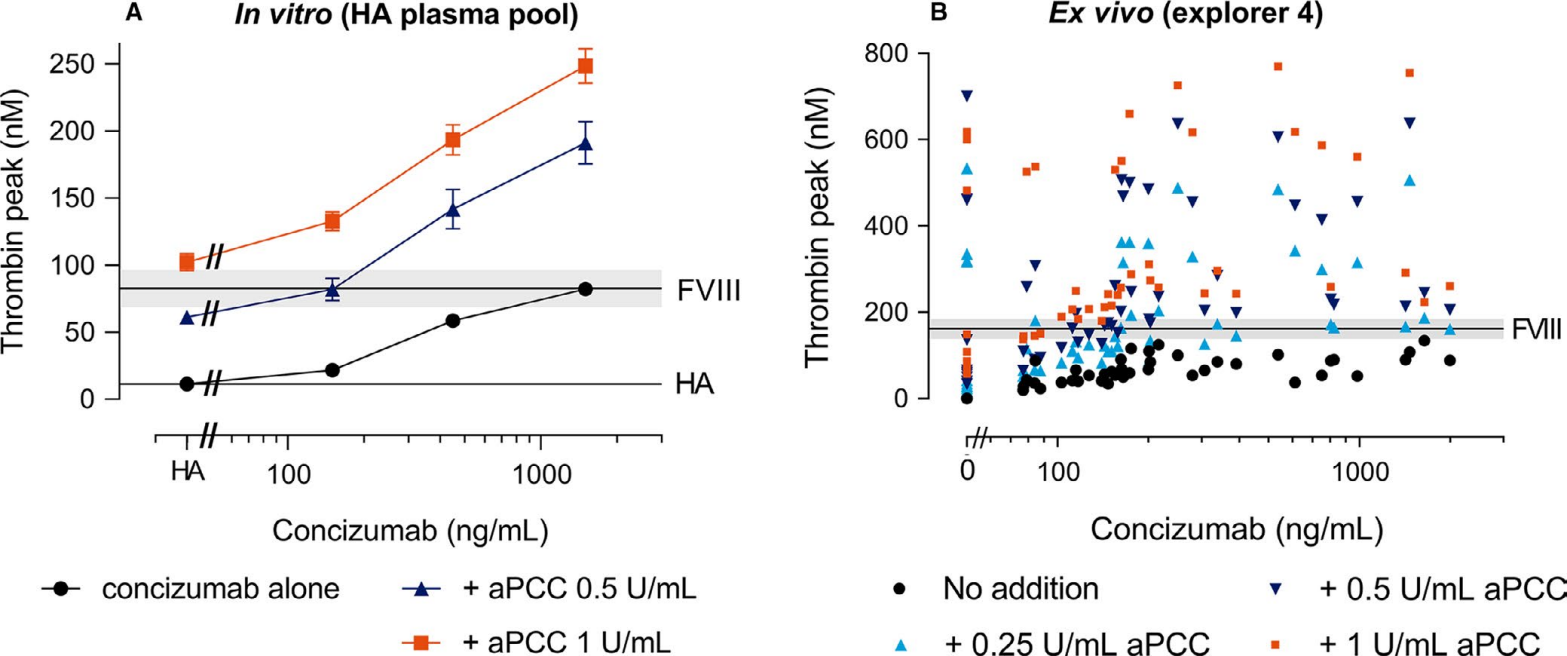
The effect of activated prothrombin complex concentrate (APCC) on thrombin generation (TG) in the absence or presence of concizumab *in vitro*, in a hemophilia A (HA) plasma pool (A) and *ex vivo*, in plasma samples from HA patients with inhibitors dosed concizumab (B). Concizumab alone or in combination with APCC at 0.5 or 1 U/ml was added to a HA plasma pool (A). APCC (0.25; 0.5 or 1 U/ml) was added to plasma samples from HA patients with inhibitors participating in the explorer4 trial at baseline before concizumab administration (data points at 0 ng/ml concizumab) and after 8, 12, and 16 weeks of daily subcutaneous concizumab administration (B). TG was measured after initiation of the assay with 1 pM tissue factor and values for the thrombin peak are depicted (A: mean ± 1 standard deviation [SD] of *n* = 3; B: single measurements of all plasma samples). Values shown in (B) are baseline‐subtracted, that is, values for pre‐concizumab samples without spiking are subtracted. The upper black horizontal line and the gray area depicts (A) the mean ± 1 SD of the thrombin peak observed upon addition of recombinant factor VIII (rFVIII) at 1 IU/ml to the HA plasma pool to reflect normal FVIII plasma level or (B) the mean and 95% confidence interval (CI) of rFVIII (1 IU/ml) added to non‐inhibitor HA plasma (explorer5 baseline samples). The bottom horizontal line (A) represents the mean ± 1 SD of the thrombin peaks observed for the HA plasma pool without spiking

APCC had a significant (*P* < .001) and concentration‐dependent effect in increasing the thrombin peak both in the absence and presence of concizumab (Figure 2A). Combining concizumab and APCC resulted in a further increased thrombin peak. In contrast to rFVIIa, the assay was very sensitive to APCC.^16^ The effect of APCC at 1 U/ml, corresponding to the plasma concentration after dosing with 100 U/kg^17^ and concizumab at 150 ng/ml resulted in a thrombin peak slightly above the upper 95% CI of the thrombin peak obtained when a normal level of rFVIII (1 IU/ml) was added to non‐inhibitor baseline plasma (explorer5). The thrombin peak obtained by adding 1 U/ml APCC was 102 ± 6 nM. By interpolation of the concentration‐response curve of concizumab in the presence of 0.5 U/ml APCC, the same thrombin peak may be reached at 238 ng/ml concizumab and 0.5 nM APCC (Figure 2A). As a concizumab concentration of 238 ng/ml is within the anticipated clinically relevant range of concizumab, the data suggest that an APCC concentration of 0.5 U/ml (50 U/kg) in the presence of concizumab would result in the same TG potential of the plasma as is obtained with 1 U/ml APCC in the absence of concizumab.

The effects of concizumab and APCC were mainly additive, with statistically significant drug‐drug interactions observed for 1 U/ml APCC added to 150–1500 ng/ml concizumab, and 0.5 U/ml APCC added to 450–1500 ng/ml concizumab (Figure S2). The additive effects of concizumab and APCC accounted for a total of 83% of the observed effects seen when combining 150 ng/ml concizumab with APCC at 1 U/ml, and 68% when combining 1500 ng/ml concizumab with APCC at 0.5 or 1 U/ml. Increased concentrations of concizumab (4500 and 15,000 ng/ml) led to only a small or no further increase in thrombin peak compared to 1500 ng/ml concizumab both in the presence and absence of APCC (Figure S3B).

#### 3.2.2 The effect of spiking patient plasma samples (explorer4) with APCC

Based on high thrombin peaks observed upon addition of APCC to HA patient plasma from the explorer5 study (data not shown), an additional, lower APCC dose of 0.25 U/ml was included in the evaluation of patient samples with HA with inhibitors from explorer4. Spiking APCC to plasma samples from patients who had received concizumab prophylaxis led to a concentration‐dependent increase in thrombin peaks both in the absence and presence of concizumab (Figure 2B; examples from individual patient samples shown Figure S7). Thrombin peaks above the 95% CI of the level obtained by adding a normal plasma level of FVIII (1 IU/ml) to non‐inhibitor plasma (explorer5) were also observed for APCC in the absence of concizumab, reflecting that the assay is very sensitive to APCC.

Evaluation of the mean and 95% CI of differences in thrombin peak increase after addition of APCC to plasma with or without concizumab showed that the overall effects of 1 U/ml APCC were statistically significantly larger in the presence of concizumab than in its absence (Figure S5), while there was no statistically significant difference observed for 0.5 and 0.25 U/ml APCC. This result indicates the presence of drug‐drug interactions (i.e., a synergistic effect) between concizumab and 1 U/ml APCC. The additive effects of concizumab and 1 U/ml APCC accounted for 80% ± 22% of the total observed effects of combining 1 U/ml APCC with concizumab, with the remaining effect corresponding to the magnitude of the drug‐drug interaction (Figure S6).

### 3.3 The effect of combining concizumab and rFVIII

#### 3.3.1 The effect of adding rFVIII to a HA plasma pool

**FIGURE 3:**
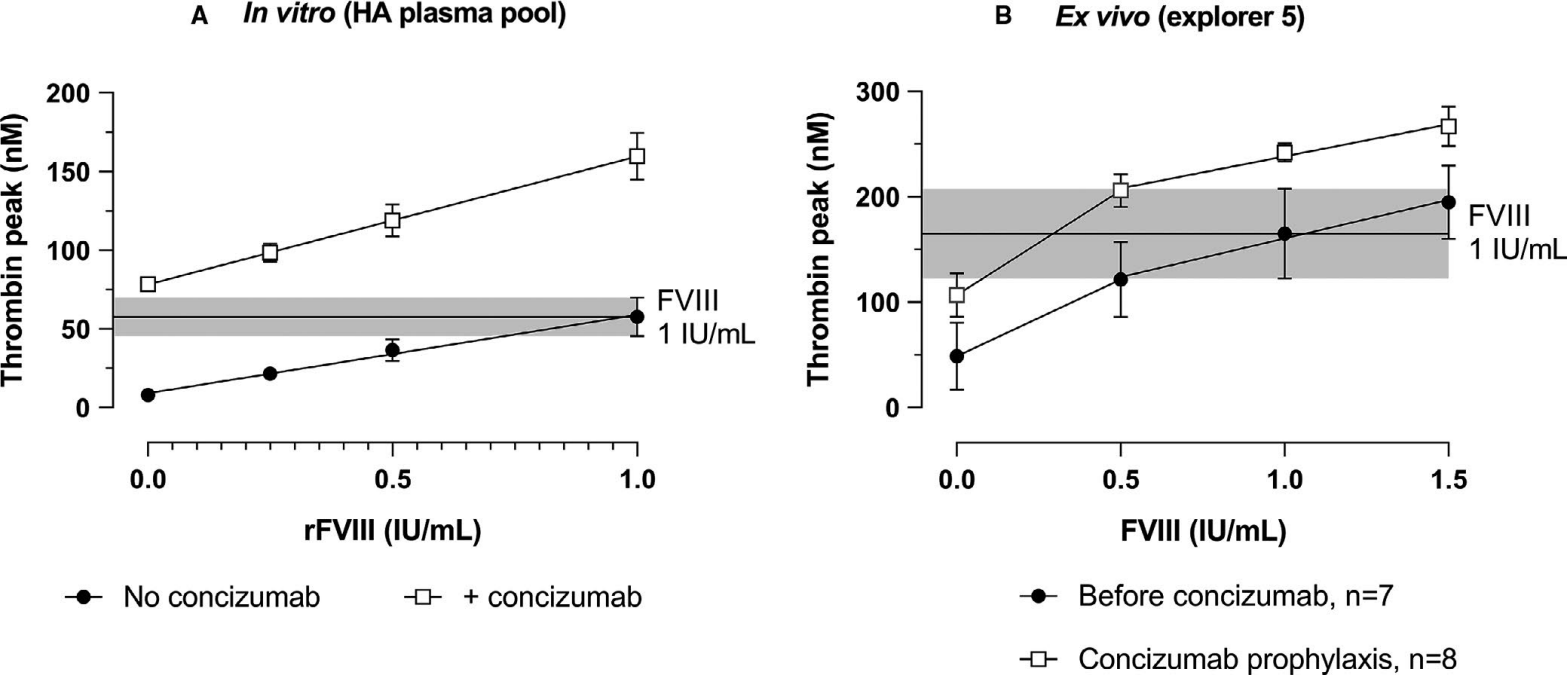
The effect of activated recombinant factor VIII (rFVIII) on thrombin generation (TG) in the absence or presence of concizumab in a hemophilia A (HA) plasma pool (A) or in HA patient plasma samples before and during concizumab prophylaxis (B). An HA plasma pool was spiked with different concentrations of rFVIII in the presence of absence of 1500 ng/ml concizumab (A, *n* = 4). rFVIII was added to plasma samples from patients with HA participating in the explorer5 trial (B). A total of 33 plasma samples from eight patients in which free tissue factor (TF) pathway inhibitor (TFPI), that is, TFPI not bound to concizumab, was below the lower limit of quantification of the assay were included together with baseline samples (before concizumab administration) from seven of these patients. TG was measured after initiation of the assay with 1 pM TF, and mean and standard deviation (SD) of values for thrombin peak are shown on the graphs. The black horizontal line and the gray area depicts the mean ± 1 SD of the thrombin peak observed upon addition of rFVIII at 1 IU/ml reflecting normal FVIII plasma

rFVIII was added to a HA plasma pool at concentrations of 0.25, 0.5, and 1 IU/ml corresponding to plasma levels after administration of 12.5, 25, and 50 IU/kg, respectively, assuming an incremental recovery of 2%. A concentration‐dependent increase in thrombin peak was observed both in the absence and presence of concizumab at 1500 ng/ml (Figure 3A) and at the higher concentrations of 4500 and 15,000 ng/ml (Figure S3C). The effect of rFVIII was significant at all concentrations of concizumab (*P* < .001). For each concentration of rFVIII, the thrombin peak increased only slightly further by increasing the concentration of concizumab from 1500 ng/ml to 4500 and 15,000 ng/ml.

The isolated effects of concizumab and rFVIII were compared to the observed effects in samples in which both concizumab and rFVIII were present. A significantly greater effect was observed for the combination of rFVIII and concizumab than for the sum of effects of rFVIII and concizumab, indicating the presence of drug‐drug interactions. The additive effects of concizumab and rFVIII accounted for between 78% and 81% (1 IU/ml rFVIII) and 89% and 94% (0.25 IU/ml rFVIII) of the total observed effects, with up to approximately 20% caused by drug‐drug interactions (Figure S8).

#### 3.3.2 The effect of adding rFVIII to HA patient plasma samples (explorer5)

rFVIII (0.5, 1, and 1.5 IU/ml) was added to 33 plasma samples from eight patients with HA who had received concizumab prophylaxis and for which free TFPI (TFPI not bound to concizumab) was below the lower limit of quantification in the assay (9.6 ng/ml). rFVIII was also added to pre‐dose samples available for seven of these patients. Thrombin peaks were higher with than without concizumab, supporting that concizumab enhances the TG potential of the plasma in the patients (Figure 3B). Similar to the results *in vitro*, addition of rFVIII increased the thrombin peak in a concentration‐dependent manner both in the absence and presence of concizumab. The effect of adding rFVIII was significant both in pre‐dose samples and in samples from the patients on concizumab prophylaxis (*P* < .0001). No statistically significant drug‐drug interaction between rFVIII and concizumab was observed in this study (*P* = .39). The thrombin peak in pre‐dose samples in which 1 IU/ml rFVIII was added, corresponding to the approximate plasma level after administration of 50 IU/kg rFVIII, was 166 ± 43 nM (mean and standard deviation of *n* = 7). In plasma samples from patients on concizumab prophylaxis, a slightly higher thrombin peak (206 ± 16 nM, *n* = 8) was observed when 0.5 IU/ml rFVIII was added, indicating that rFVIII dosing may be reduced by ~50% in the presence of concizumab to obtain the same TG potential of the plasma as with a full rFVIII dose in the absence of concizumab.

### 3.4 The effect of combining concizumab and rFIX

#### 3.4.1 Spiking of HB plasma pool with rFIX in the absence or presence of concizumab

**FIGURE 4:**
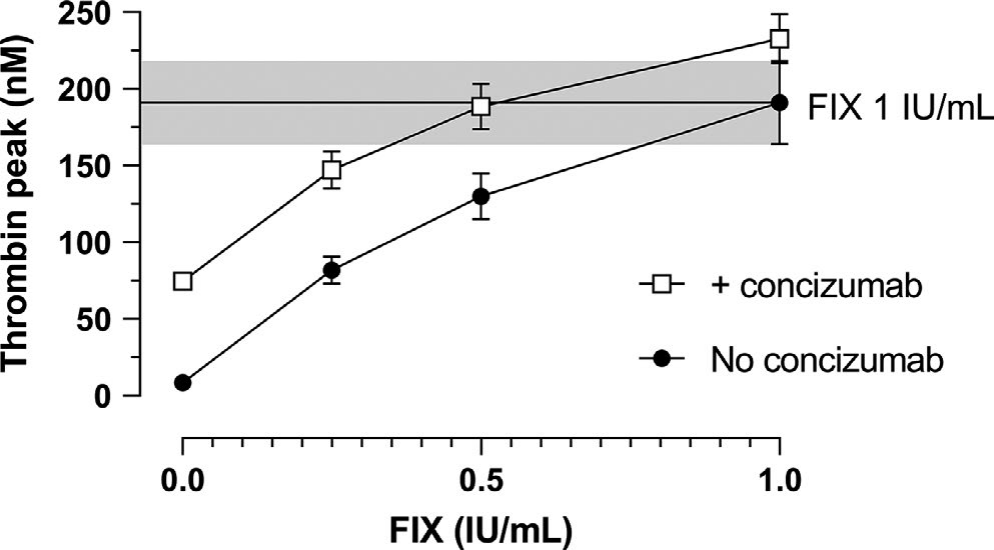
The effect of recombinant factor IX (rFIX) on thrombin generation (TG) in a hemophilia B (HB) plasma pool in the absence and presence of concizumab. rFIX was added to an HB plasma pool in the absence or presence of concizumab at 1500 ng/ml, and TG was measured after initiating the assay with 1 pM tissue factor. Mean and standard deviation (SD; *n* = 3) of values for thrombin peak are depicted. The black line and gray area represent the mean ± 1 SD of values seen upon the addition rFIX at 1 IU/ml to the HB plasma pool to reflect normal FIX plasma levels without concizumab

rFIX at concentrations of 0.25, 0.5, and 1.0 IU/ml corresponding to approximate maximal plasma level after dosing 12.5−25, 25−50, and 50−100 IU/kg, respectively, assuming incremental recovery of 1% to 2%, was added to a HB plasma pool alone or combined with concizumab (Figure 4). The addition of rFIX resulted in a significant, concentration‐dependent increase of thrombin peak (*P* < .001), both in the absence and presence of concizumab at 1500 ng/ml. Likewise, concizumab increased the thrombin peak in the HB plasma at all rFIX concentrations (*P* < .001). Increasing concizumab concentration from 1500 to 4500 ng/ml and 15,000 ng/ml had no further effect on the thrombin peak at any of the rFIX concentrations (Figure S3D), supporting that plasma TFPI is saturated with concizumab at 1500 ng/ml. The majority of the observed effects of rFIX and concizumab were additive (Figure S9); however, significant negative drug‐drug interactions were observed for the combination of 1 IU/ml rFIX with concizumab at all concentrations and for 0.5 IU/ml rFIX combined with 15,000 ng/ml concizumab. The magnitude of this negative drug‐drug interaction was maximally 10% of the total observed effects, indicating that a reduced effect of rFIX by up to 10% may be expected in the presence of concizumab.

The thrombin peak obtained upon adding 1.0 IU/ml rFIX to plasma without concizumab was similar to the thrombin peak in the presence of concizumab and 0.5 IU/ml rFIX (191 ± 27 nM and 189 ± 15 nM, respectively). This suggests that in the presence of concizumab, a two‐fold reduced dose of rFIX would be sufficient to obtain the same plasma TG potential as with 1.0 IU/ml rFIX in the absence of concizumab.

## 4 DISCUSSION

With the development of novel, non‐replacement therapies for the treatment of hemophilia, it is important to investigate the concomitant use of procoagulant agents, as there is a potential risk of thrombotic complications. In particular, it is important to be aware if strong synergistic effects (i.e., effects larger than the sum of the effects of the individual drugs as a result of drug‐drug interaction) can be expected in the case of treatment of bleeding episodes in patients on prophylactic therapy with novel, non‐replacement therapies. In the present analysis, using a TG assay, we investigated the *in vitro* effect of the bypassing agents rFVIIa and APCC in a HA plasma pool spiked with concizumab and *ex vivo*, in samples from patients treated prophylactically with concizumab in the phase 2 trials. In additional analyses, the effect of rFVIII in pooled HA plasma spiked with concizumab and patient plasma from HA patients on concizumab prophylaxis in the phase 2 non‐inhibitor trial was evaluated. Finally, the effects of rFIX and concizumab were evaluated in pooled HB plasma. Potential drug‐drug interactions between concizumab and rFVIIa, APCC, rFVIII, and rFIX were assessed by comparing the increase in thrombin peak upon the addition of rFVIIa/APCC/rFVIII or rFIX to plasma with concizumab relative to the increase in thrombin peak upon addition of rFVIIa, APCC, FVIII, or FIX to plasma without concizumab plus the isolated effect of concizumab.

The addition of rFVIIa, APCC, or rFVIII to a HA plasma pool or to samples from patients with HA and inhibitors (explorer4; rFVIIa and APCC) or patients with HA without inhibitors (explorer5; rFVIII) increased thrombin peaks both in the absence and presence of concizumab. Likewise, an increase in thrombin peak was seen when adding rFIX to a HB plasma pool both in the absence and presence of concizumab. For both the combination of concizumab with APCC or rFVIIa, the majority of the observed effects on thrombin peaks was additive. Similarly, the effect of rFVIII and concizumab was mainly additive, accounting for ≥80% of the observed effect on a HA plasma pool and for all the observed effects upon spiking rFVIII to plasma from HA patients on concizumab prophylaxis. Overall, drug‐drug interaction effects between concizumab combined with either rFVIIa or APCC were found to be smaller than the isolated effects of concizumab. This small synergistic effect is consistent with the mechanism of action of concizumab,^6^ as well as those of rFVIIa, APCC, and rFVIII, and can be explained by the effect of concizumab on TFPI‐mediated inhibition of FXa generated through rFVIIa and—in the case of non‐inhibitor plasma—the FVIIIa/FIXa complex. FXa would normally bind to TFPI, initiating a negative feedback loop on its own production via TFPI/FXa binding to and inhibiting TF/FVIIa. However, when TFPI is bound to concizumab, FXa inhibition is abolished resulting in a more sustained FXa activity. APCC contains FVII, FVIIa, FX, FIX, FII (prothrombin); some FVIII antigen; and traces of FIXa and FXa, leading to increased FX and prothrombin activation.^17^, ^18^, ^19^ TFPI inhibits FXa and early forms of the prothrombinase complex (FXa in complex with FXa‐activated or platelet‐derived FVa), and by blocking TFPI, concizumab could counteract the inhibition of FXa and enhance the effect conferred by APCC. The additive effects of rFIX added to a HB plasma pool spiked with concizumab were greater than the total observed effect for the highest concentrations of rFIX. This observation reflects negative drug‐drug interactions, that is, an approximately 10% reduced effect when concizumab was combined with rFIX. The reason behind this relatively small negative drug‐drug interaction is not clear and will require further investigation. It should be noted that the present studies are based on *in vitro* and *ex vivo* assay data that only include the plasma compartment, meaning that the data generated do not fully reflect the situation *in vivo* in which the majority of TFPI is located on the endothelium. Furthermore, for individual patients, a certain response in the TG assay may not necessarily translate into clinical outcome. Therefore, the assay may not be suitable for monitoring of individual patients. Additional clinical experience is needed to allow for any recommendations on the use of the TG assay for monitoring.

The combination of concizumab and rFVIIa was previously evaluated *in vivo* in a bleeding model in rabbits with antibody‐induced hemophilia and in a safety study in cynomolgus monkeys.^20^ No signs of additive or synergistic effects of rFVIIa and concizumab were observed in the rabbit model, possibly because the maximal effect was already achieved by individual treatment with concizumab or rFVIIa. Consistent with the clinical phase 1 and 2 data,^7^, ^21^, ^22^ treatment with concizumab resulted in increased thrombin‐antithrombin (TAT) and D‐dimer levels in monkeys, indicating activation of coagulation and fibrinolytic pathways. An additional increase in TAT and D‐dimers was observed after administration of three doses of rFVIIa to animals receiving concizumab daily for 4 weeks, but without concomitant formation of thrombi or other signs of exaggerated coagulation.

In a phase 3 trial of the FVIIIa‐mimicking bispecific antibody emicizumab (Hemlibra^®^; F. Hoffmann‐La Roche AG), TEs and thrombotic microangiopathy cases occurred when patients with inhibitor‐complicated hemophilia used APCC (>100 U/kg/24 h) to treat breakthrough bleeds.^23^ *In vitro* experiments showed that APCC in combination with a sequence‐identical analog (SIA) of emicizumab at clinically relevant concentrations resulted in excessive TG.^16^, ^19^ The emicizumab SIA plus APCC at 0.5 U/ml resulted in peak thrombin levels that were 17‐fold higher versus the emicizumab SIA alone. Hypercoagulability was demonstrated with as little as 0.25 U/ml APCC, with the synergistic effect being driven primarily by the FIX component of APCC. In the same study, the emicizumab SIA plus rFVIIa (1.75 μg/ml corresponding to 35 nM, i.e., above the plasma concentration of 19 ± 3 nM achieved after administration of 90 µg/kg rFVIIa^15^) did not lead to any excessive increase in TG, but resulted in a 1.8‐fold increase in the peak thrombin levels. By comparison, it should be noted that the present study using the same TG assay did not demonstrate such an exaggerated effect for the combination of concizumab and APCC (or rFVIIa).

Fitusiran, an RNA interference therapeutic targeting antithrombin, is under clinical investigation in patients with HA and HB with or without inhibitors. In an *in vitro* analysis, addition of rFVIIa and APCC to severe HA/HB plasma immunodepleted of antithrombin had an additive effect.^24^ In a more recent *ex vitro* investigation in plasma samples from patients with HA with or without inhibitors collected before and after treatment with fitusiran, it was demonstrated that the addition of rFVIIa or APCC further enhanced the initial increase in TG potential observed following the fitusiran‐induced reduction in antithrombin. The authors concluded that reduced doses of bypassing agents may be sufficient to achieve hemostasis in patients experiencing breakthrough bleeds during fitusiran therapy.^25^ Both the fitusiran and emicizumab studies used the same TG assay as used in the present study; however, data generated in different laboratories may not be directly comparable as this type of assay is susceptible to laboratory‐to‐laboratory variation allowing only a qualitative comparison.

Following the implementation of new safety measures and guidelines aiming to minimize the risk of TEs in patients treated with concizumab, the phase 3 explorer7 and explorer8 concizumab trials were re‐initiated in September 2020.^13^ Protocol adjustments include specific recommendations for the treatment of mild and moderate breakthrough bleeding episodes as the main risk mitigation. Treatment recommendations for bleeds mandate a cautious approach when administering additional hemostatic agents in patients on concizumab prophylaxis, using the lowest approved dose of any replacement product or bypassing agent and with the time interval between two doses, if relevant, not shorter than stated on the label. The mitigation includes closer oversight of treatment for breakthrough bleeds and potential concizumab dose adjustments to ensure that patients are exposed to enough concizumab to prevent bleeding, while avoiding unnecessary exposure beyond what is needed to saturate and inhibit endogenous TFPI.^12^

A fine balance is required between cautious dosing with hemostatic agents to avoid TEs while at the same time avoiding undertreatment. The present study demonstrated that rFVIIa, APCC, and rFVIII were fully active in enhancing plasma TG potential in the presence of concizumab and suggested an up to approximately 25% additional effect conferred by drug‐drug interaction. For rFIX, the data in the present study did not point to any exaggerated TG in HB plasma spiked with concizumab. Together, these *in vitro* data support the use of rFVIIa, APCC, rFVIII, and rFIX for the treatment of mild and moderate breakthrough bleeds in patients on concizumab prophylaxis using the lowest approved doses known to be effective (i.e., lowest approved dose as per label) in order to alleviate potential safety concerns, while maintaining the hemostatic effect needed to treat the bleeds.

## CONFLICTS OF INTEREST

All authors either are or were employees of Novo Nordisk A/S at the time this study was conducted.

## AUTHOR CONTRIBUTIONS

M. Kjelgaard‐Hansen and I. Hilden planned the rFVIIa and APCC studies and M. Kjelgaard‐Hansen was responsible for the rFVIIa and APCC plasma pool study and analysis of samples from non‐inhibitor patients. M. Kjalke completed the rFVIIa and APCC studies, planned the rFVIII and rFIX studies, and analyzed the data. S. Andersen performed the statistical analysis. All authors contributed to the writing and review of the manuscript and approved the final version.

## Supporting information

Fig S1

Fig S2

Fig S3

Fig S4

Fig S5

Fig S6

Fig S7

Fig S8

Fig S9

Video S1

Supplementary Material
