# Supplementary figures and images for: Thrombin generation potential in the presence of concizumab and rFVIIa, APCC, rFVIII, or rFIX: In vitro and ex vivo analyses
Source: J Thromb Haemost. 2021 May 6;19(7):1687–96. doi: 10.1111/jth.15323 (PMC8360123; doi:10.1111/jth.15323)

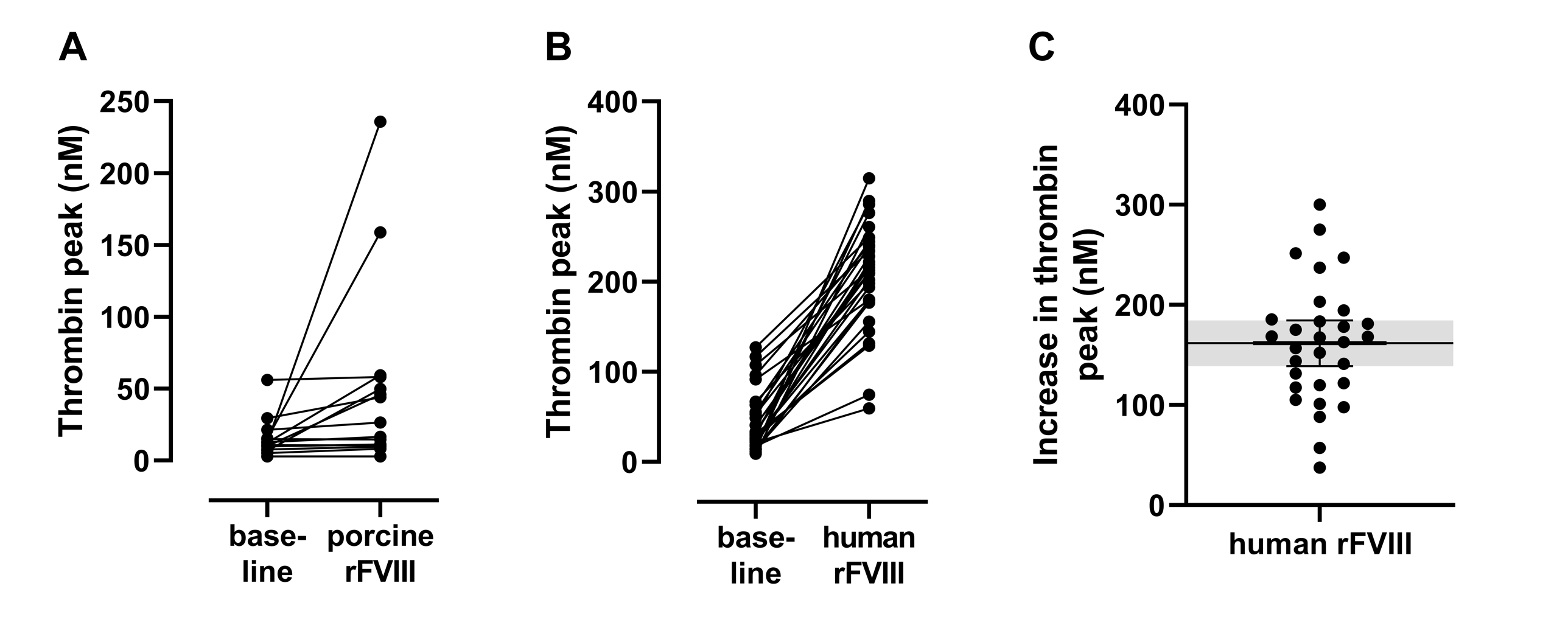

Supplement: Supplementary file 1 — Fig S1 [file JTH-19-1687-s010.tif]

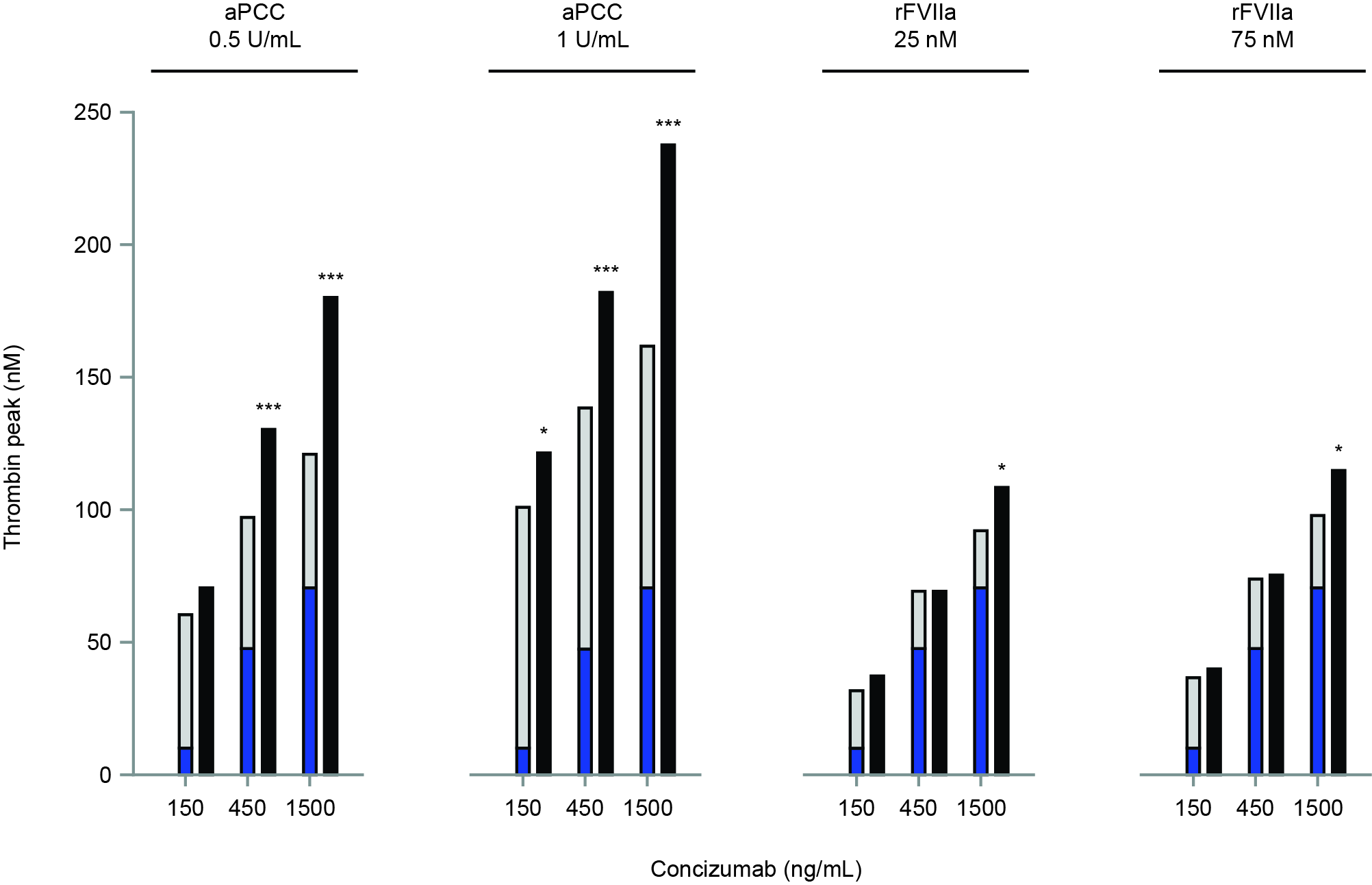

Supplement: Supplementary file 2 — Fig S2 [file JTH-19-1687-s008.tif]

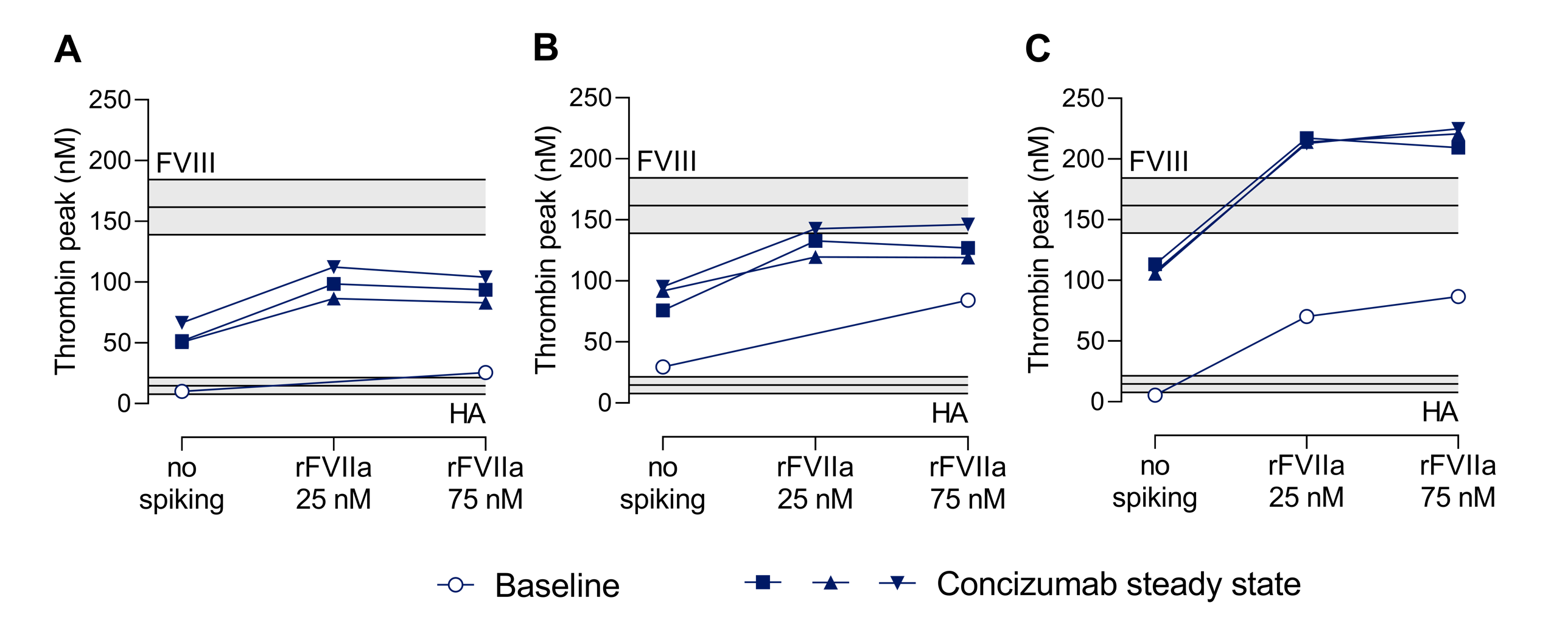

Supplement: Supplementary file 4 — Fig S4 [file JTH-19-1687-s004.tif]

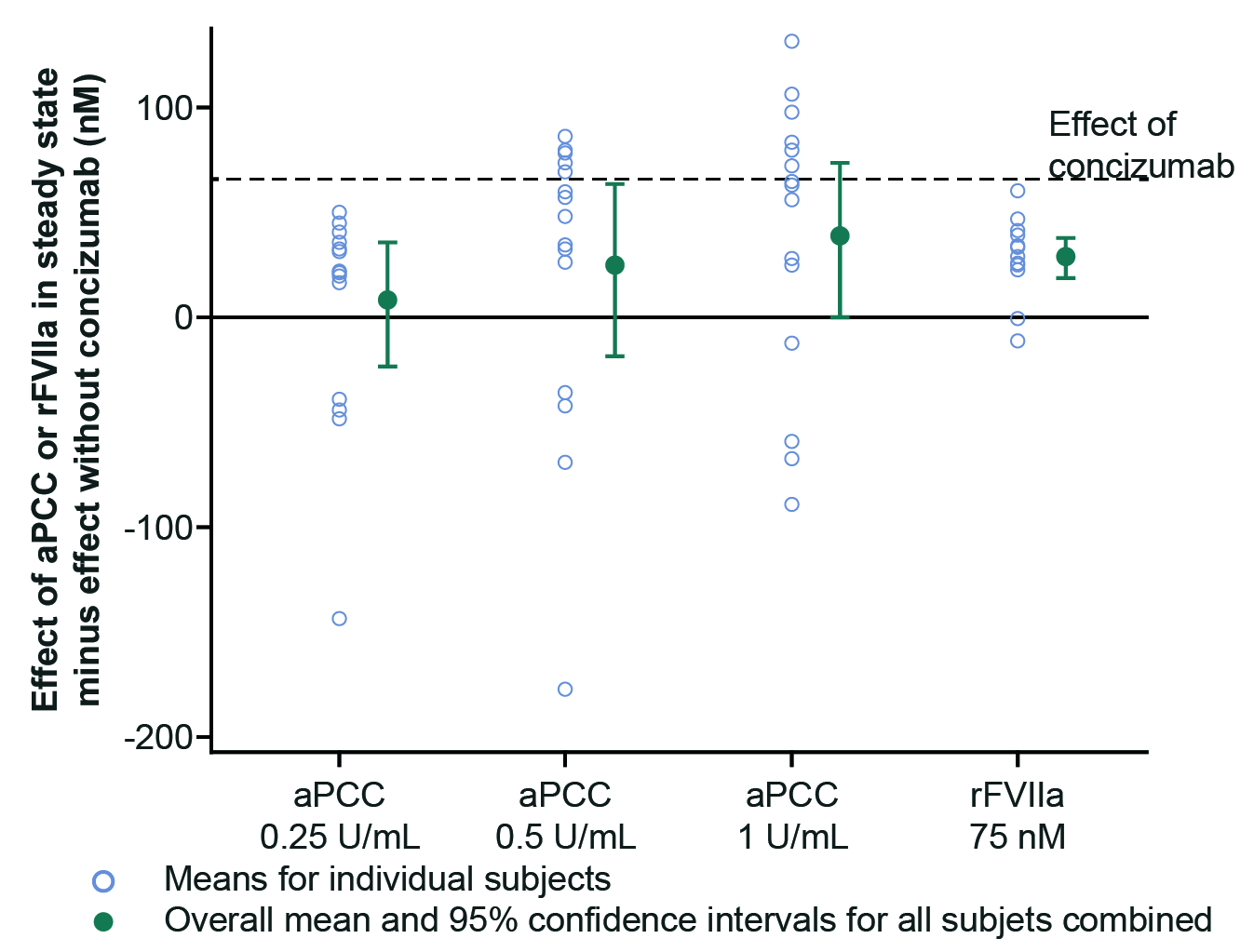

Supplement: Supplementary file 5 — Fig S5 [file JTH-19-1687-s011.tif]

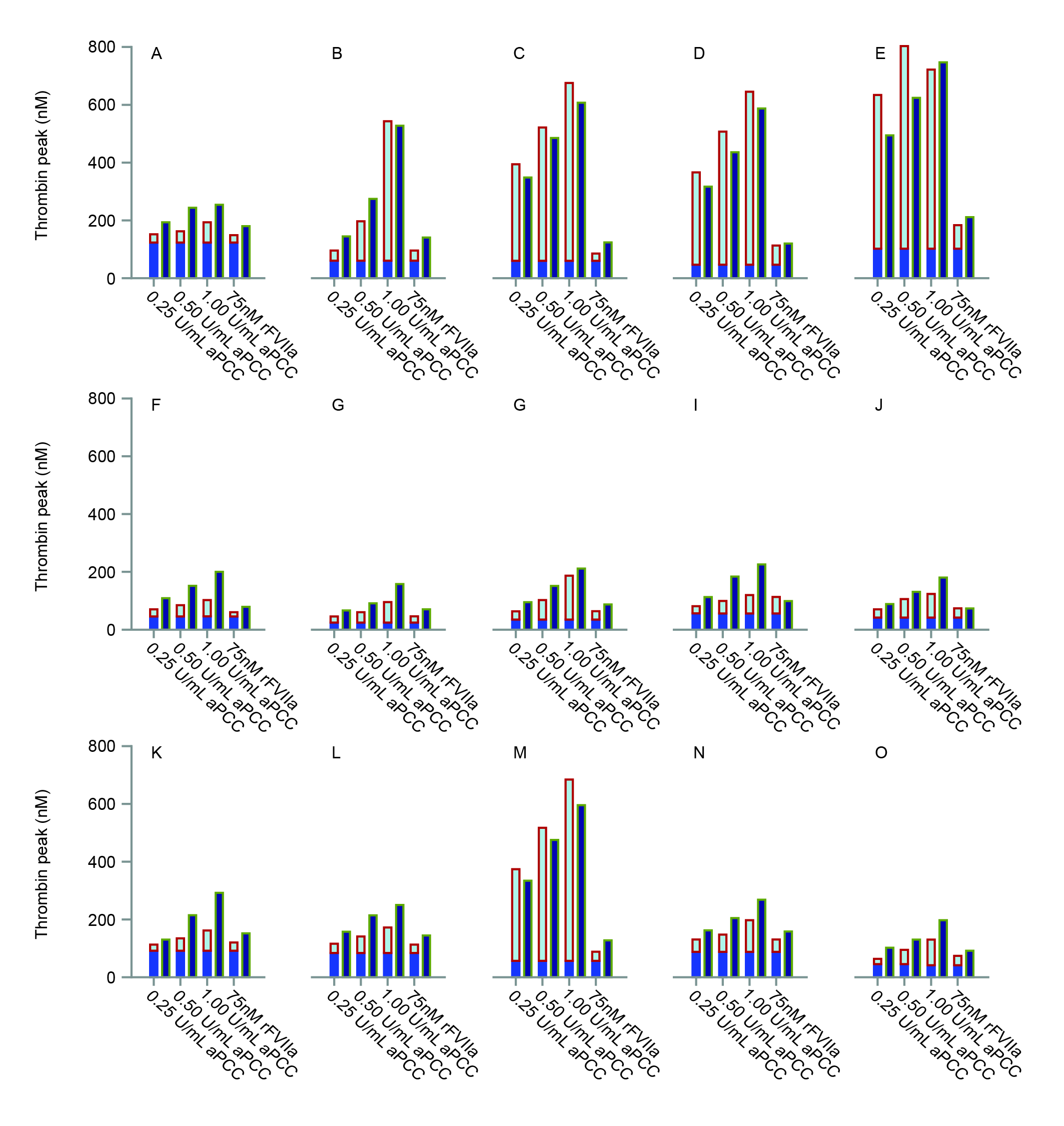

Supplement: Supplementary file 6 — Fig S6 [file JTH-19-1687-s003.tif]

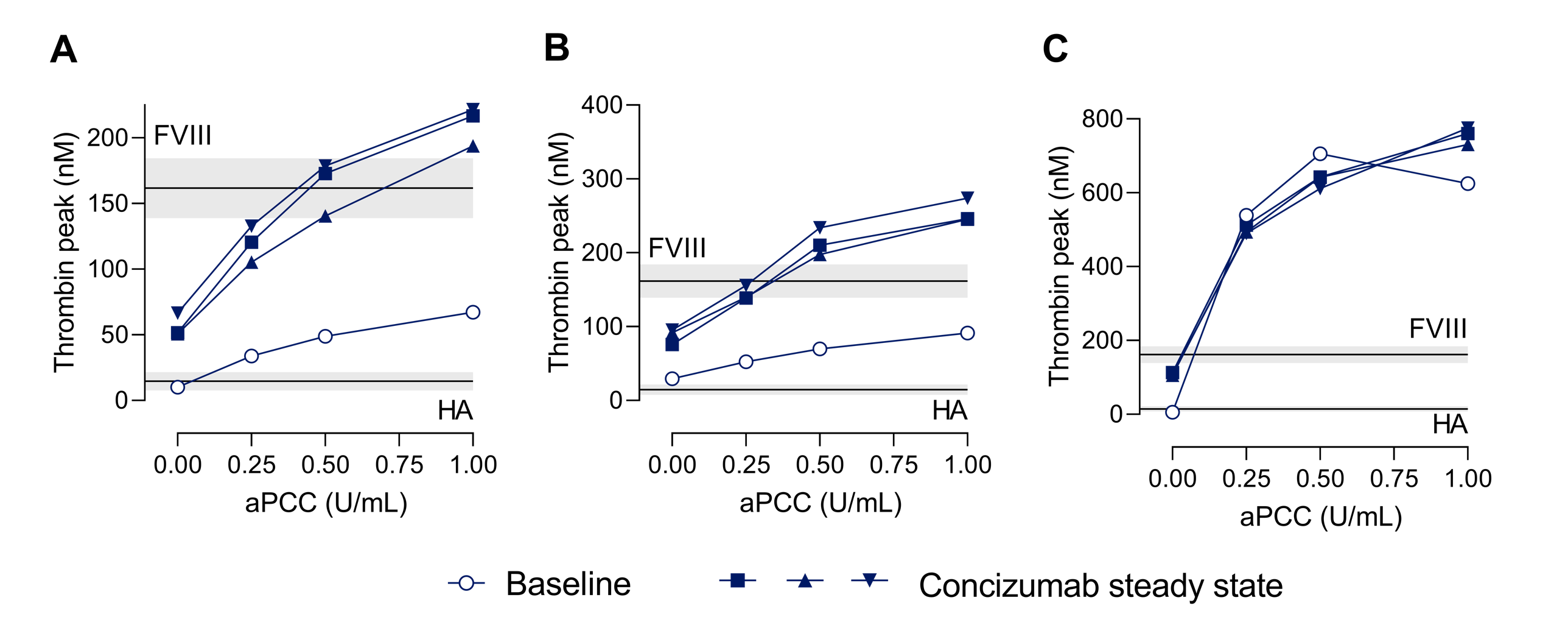

Supplement: Supplementary file 7 — Fig S7 [file JTH-19-1687-s006.tif]

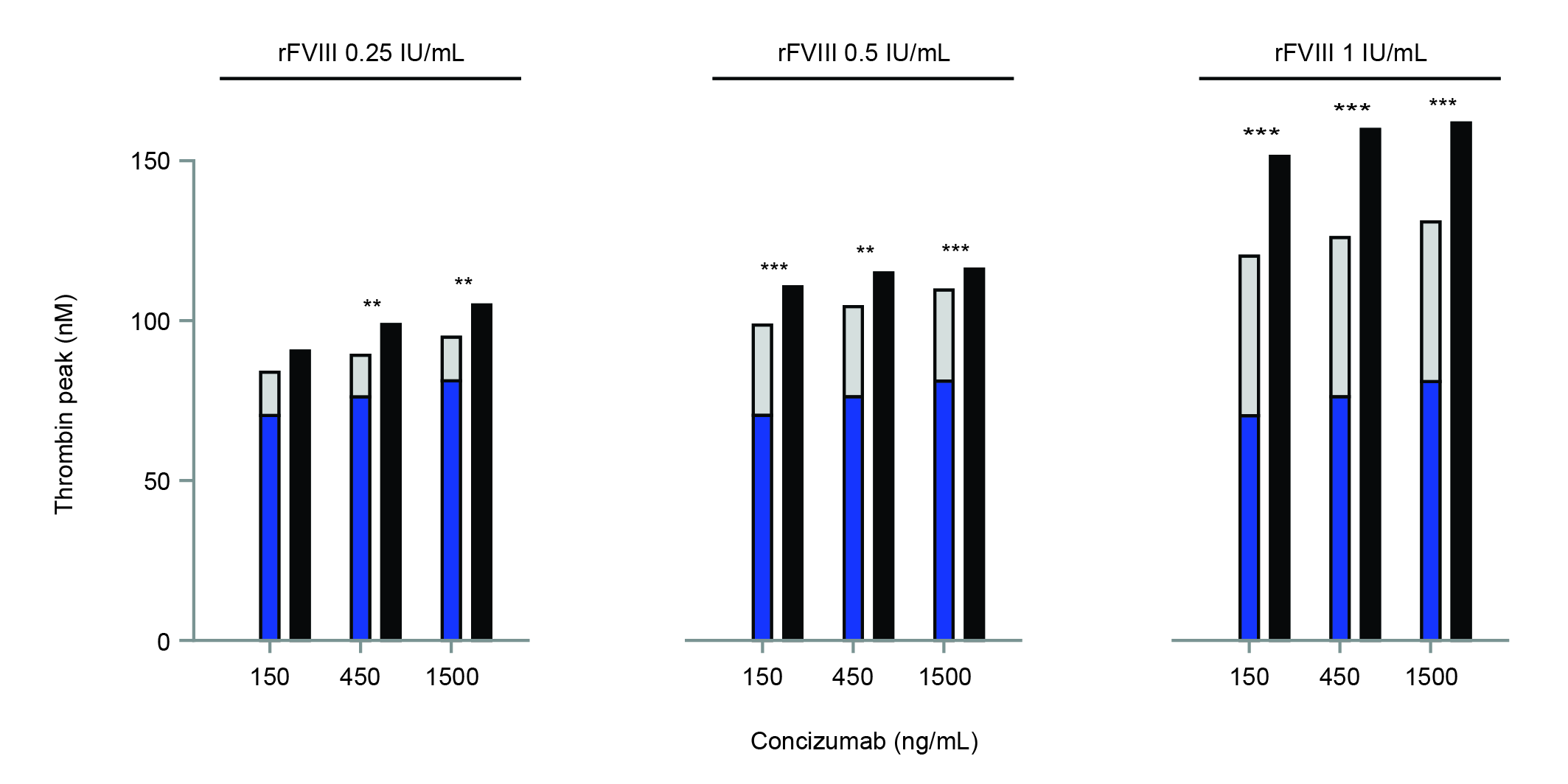

Supplement: Supplementary file 8 — Fig S8 [file JTH-19-1687-s007.tif]

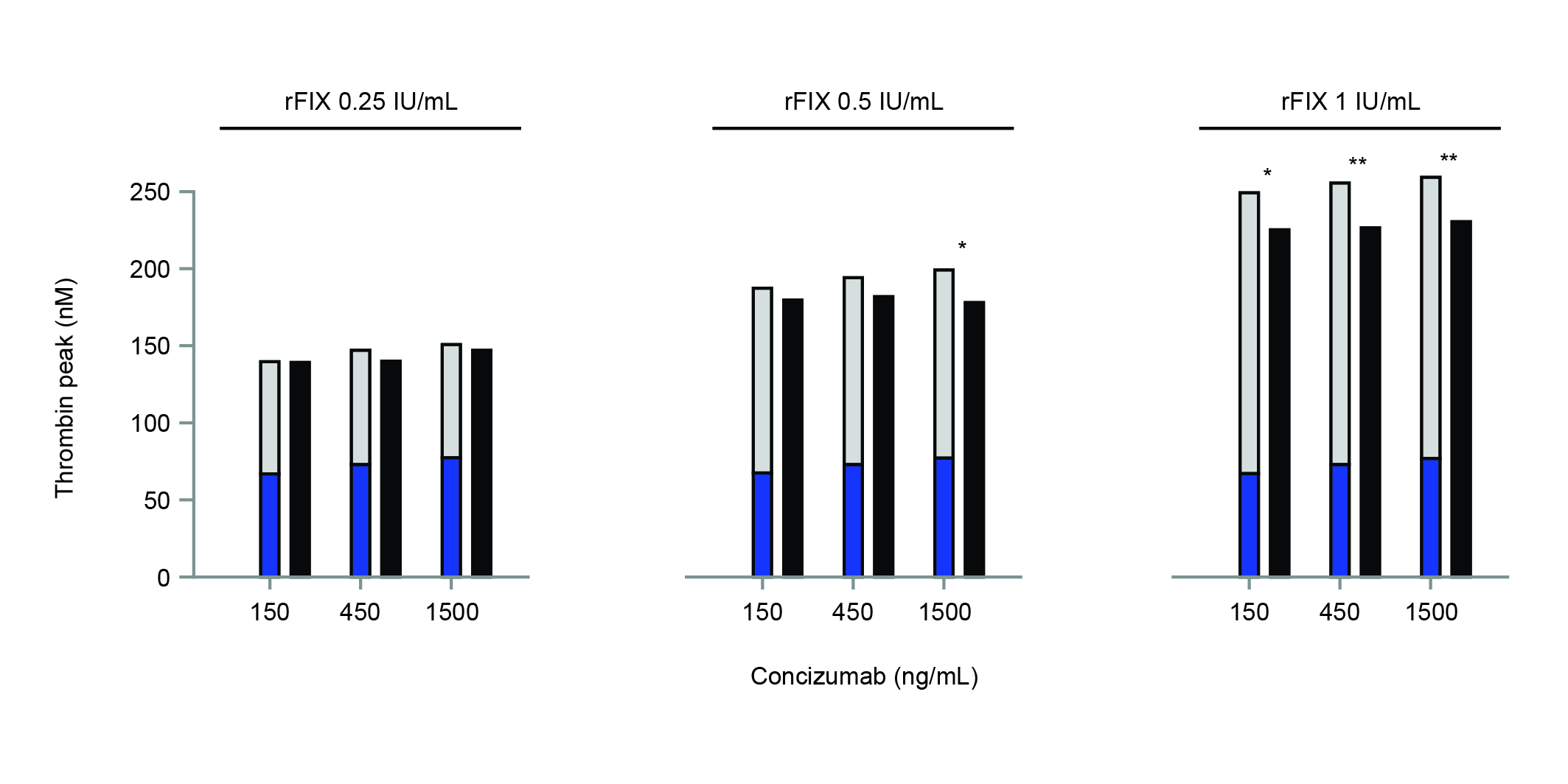

Supplement: Supplementary file 9 — Fig S9 [file JTH-19-1687-s002.tif]
