# Supplementary material for: Thrombin generation potential in the presence of concizumab and rFVIIa, APCC, rFVIII, or rFIX: In vitro and ex vivo analyses
Source: J Thromb Haemost. 2021 May 6;19(7):1687–96. doi: 10.1111/jth.15323 (PMC8360123; doi:10.1111/jth.15323)
Supplement: Supplementary file 11 — Supplementary Material [file JTH-19-1687-s005.docx]

**SUPPORTING INFORMATION**

**Statistical Methods**

***Analysis of the effect of activated recombinant factor VII (rFVIIa) and activated prothrombin complex concentrate (aPCC) in plasma samples from hemophilia A patients with (explorer4) and without (explorer5) inhibitors***

In order to analyze potential drug-drug interactions between rFVIIa (at concentrations of 25 or 75 nM) or aPCC (at 0.25, 0.5 or 1 U/mL) and concizumab, the following statistical model was applied to calculate the effects seen in each patient separately:

$$Y_{vt}= \mu+\alpha_{ss}*SS+ R_{v}+t*SS+ \varepsilon_{vt}$$

Therein, Y_vt_ reflects the measured thrombin peak, v the respective visit and t the respective treatment (i.e., no spiking or spiking at the different concentrations of aPCC and rFVIIa described earlier). The variable μ was applied as a measure of the general level measured, and α describes the effect on the measurement at steady state (mean of values for visit 5, 6 and 7 samples). SS is a binary variable differentiating between steady state (SS=0, visit 1) or thereafter (SS=1, visit 5, 6 and 7). The term t * SS describes the interaction between treatment and steady state. R_v_ takes into account a random effect of a visit and ε_vt_ accounts for the residual error.

**Results and Discussion**

***Thrombin generation potential in the presence of recombinant factor VIII (rFVIII)***

A reference to the thrombin generation potential in individual samples from HA patients with inhibitors (explorer4) was sought to be established upon addition of porcine rFVIII to baseline samples. An increase in thrombin peaks was observed in some samples, but not in all **(Supplementary Figure 1A)**. This lack of effect on thrombin generation in some samples may be attributed to the presence of anti-FVIII inhibitory antibodies cross-reacting with porcine rFVIII. The obtained measurements could therefore not be used to quantify the thrombin generation potential in the plasma conferred by rFVIII. Instead, human rFVIII was added to non-inhibitor HA plasma from explorer5 (n=30). This resulted in an increase in the thrombin peak in all plasma samples, albeit to a variable extent (**Supplementary Figure 1B**). The mean and 95% confidence interval (CI) of the increase in thrombin peak (**Supplementary Figure 1C**) was used to define the range for normal FVIII level in subsequent spiking experiments with rFVIIa and aPCC.

Please refer to the respective sections in the main manuscript for details on Supplementary Figures 2–9.

**Supplementary Figure 1.** Thrombin generation by porcine and human rFVIII in hemophilia A patient plasma samples.

**
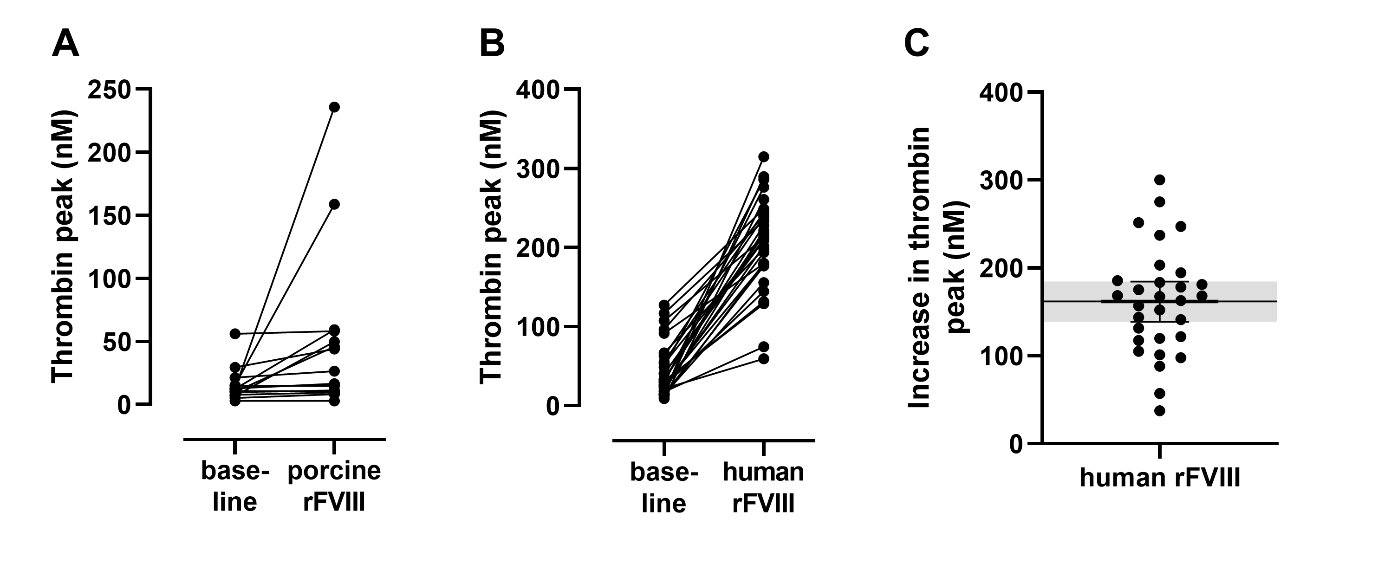
**

Porcine recombinant factor VIII (rFVIII) was added to baseline (pre-concizumab) plasma samples from inhibitor patients (explorer4, n=16) to 1 U/mL (A) and human rFVIII (1 IU/mL, reflecting normal FVIII activity level) was added to plasma samples from hemophilia A (HA) patients without inhibitors (explorer 5, n=30) (B). Thrombin generation was measured after initiating the assay with 1 pM tissue factor (TF) and values for thrombin peak are depicted. The baseline thrombin peak in each plasma sample in panel B was subtracted from the thrombin peak in the presence of human rFVIII and the mean and 95% confidence interval (CI) values were calculated and shown as the black line and grey area in panel C.

**Supplementary Figure 2.** Magnitude of additive effects relative to total observed effects on thrombin peak by concizumab and aPCC or rFVIIa spiked *in vitro* in a hemophilia A plasma pool.


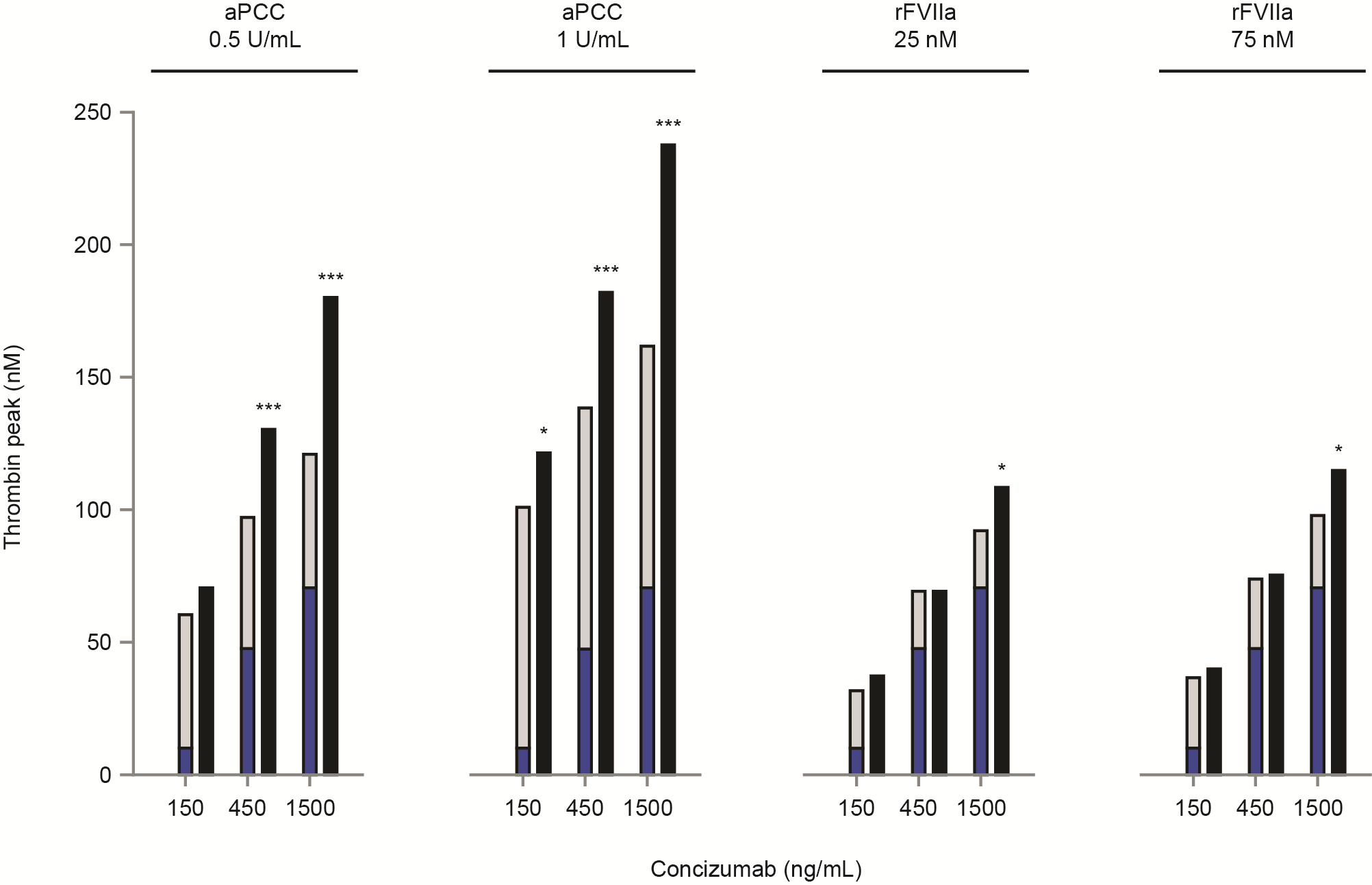


The lower part of the left columns in each grouped pair of columns (blue) represents the isolated effect of concizumab, while the upper part of the left columns (grey) represents the isolated effect of activated prothrombin complex concentrate (aPCC) or activated recombinant factor VII (rFVIIa). Thus, each left column (blue and grey part together) represents the additive effect of concizumab and either aPCC or rFVIIa. The right column in each group (black) corresponds to the observed effect of combining concizumab with aPCC or rFVIIa. The effect due to drug-drug interactions is thus represented by the difference in height of the two columns in each group. Data are mean of n=3. Levels of significance for the comparison of observed combined effects with added isolated effects were defined as follows: *p<0.05, ***p<0.001.

**Supplementary Figure 3.** No or only minor effect on thrombin peak by increasing concizumab above 1,500 ng/mL alone or in combination with rFVIIa, aPCC, rFVIII or rFIX.

**
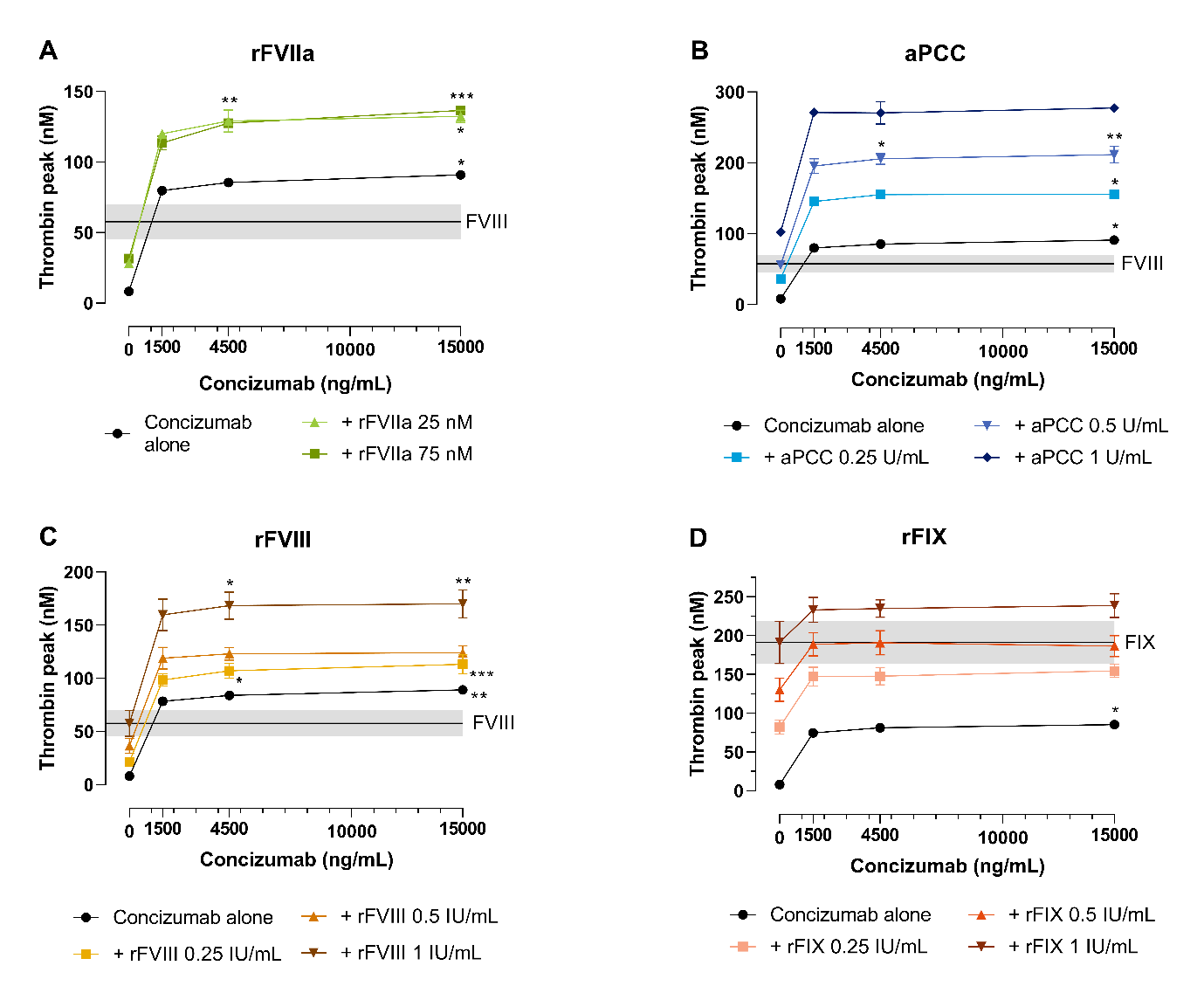
**

Concizumab (1,500–15,000 ng/mL) was added either alone or in combination with (A) activated recombinant factor VII (rFVIIa), (B) activated prothrombin complex concentrate (aPCC), or (C) recombinant factor VIII (rFVIII) to a hemophilia A (HA) plasma pool, or in combination with recombinant factor IX (rFIX) to a hemophilia B (HB) plasma pool (D). Thrombin generation was measured for all combinations after initiation of the assay with 1 pM tissue factor (TF). Values for thrombin peak (mean and standard deviation [SD] of n=3 A, B and D) or n=4 (C) are shown on the graphs. The black line and grey area represent the mean ± 1 standard deviation of the effect seen by adding (A-C) rFVIII to normal FVIII activity level (1 IU/mL) or (D) rFIX to normal FIX activity level (1 IU/mL). Significant different thrombin peaks as compared to the thrombin peak with concizumab at 1,500 ng/mL was observed when noted with *p<0.05, ** p<0.01 or *** p<0.001. There were no significant differences between thrombin peak with 4,500 ng/mL and 15,000 ng/mL concizumab in any of the combinations with rFVIIa, aPCC, rFVIII or rFIX.

**Supplementary Figure 4.** Examples of thrombin peaks after spiking plasma samples from three patients with hemophilia A and inhibitors (concizumab explorer4 trial) with rFVIIa.

*
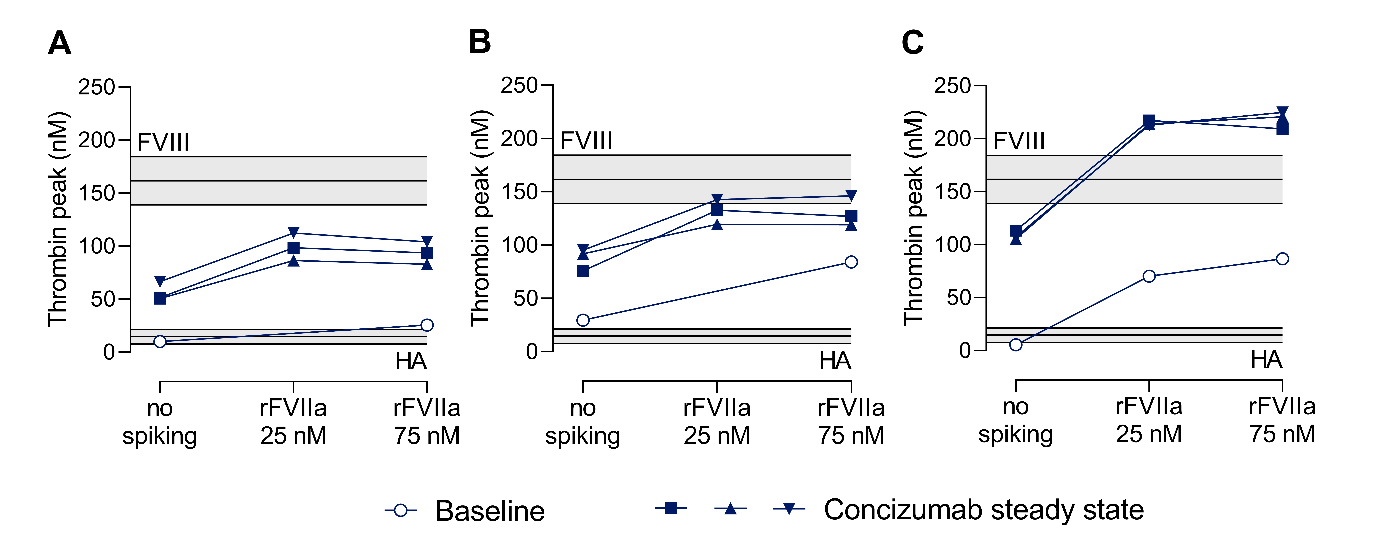
*

Activated recombinant factor VII (rFVIIa) 25 and 75 nM was added to baseline plasma samples before concizumab administration and plasma samples obtained at steady state (visits 5, 6, and 7 after 8, 12 and 16 weeks daily concizumab administration, respectively), and thrombin generation measured after initiating the assay with 1 pM TF. The area marked ‘FVIII’ represents mean and 95% confidence interval (CI) of thrombin peak after addition of recombinant factor VIII (rFVIII) to non-inhibitor plasma (from the explorer5 trial) to normal FVIII level (1 IU/mL). The area marked ‘HA’ corresponds to mean and 95% CI of thrombin peak in baseline samples in patients with hemophilia A (HA) and inhibitors (explorer4).

**Supplementary Figure 5.** Difference in thrombin peak increase after addition of aPCC or rFVIIa to hemophilia A plasma with or without concizumab.


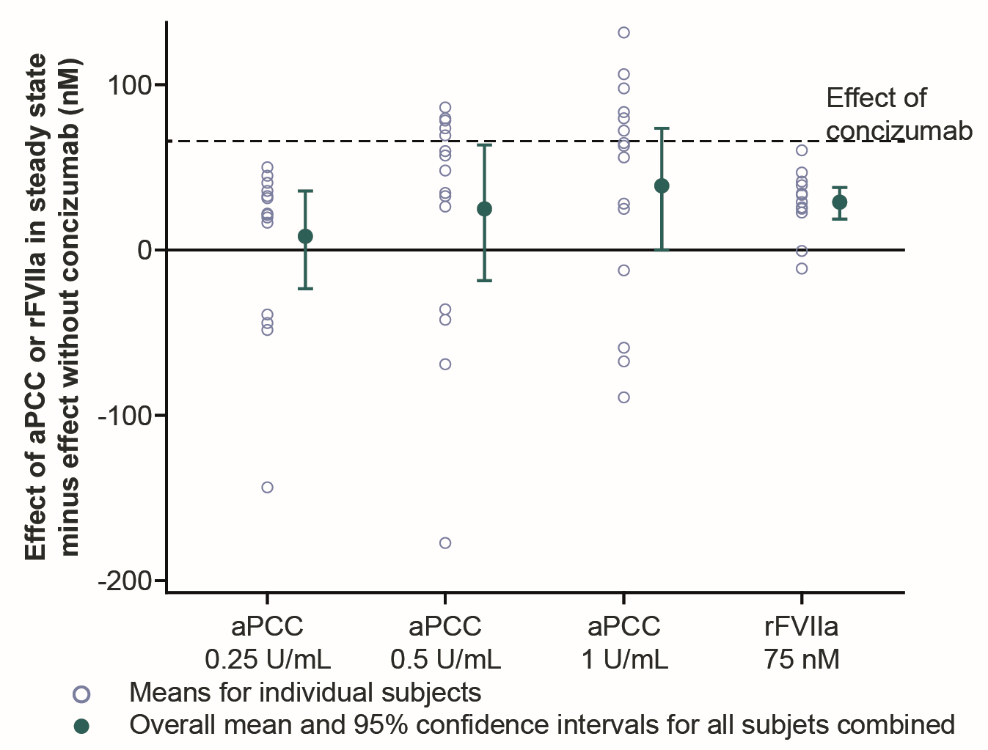


The differences in thrombin peak increase observed upon addition of either 75 nM activated recombinant factor FVII (rFVIIa) or activated prothrombin complex concentrate (aPCC) at 0.25, 0.5 or 1 U/mL to explorer4 patient plasma during concizumab prophylaxis relative to the increase observed upon addition of rFVIIa or aPCC to baseline samples before concizumab administration. Data for individual patients are shown as open circles and overall mean and 95% confidence intervals (CI) are shown as closed circles (n=15). The lower concentration of 25 nM rFVIIa was excluded from this analysis due to insufficient plasma sample availability. The mean increase in thrombin peak with concizumab is represented by the dotted line for comparison. A significantly larger effect of aPCC or rFVIIa in the presence of concizumab relative to the effect in the absence of concizumab was confirmed for 75 nM aPCC and for 75 nM rFVIIa when the 95% CI did not cross 0.

**Supplementary Figure 6.** Magnitude of additive effects on thrombin peak of concizumab and aPCC or rFVIIa relative to the total observed effect in hemophilia A plasma samples (*ex vivo* samples from the explorer4 trial).


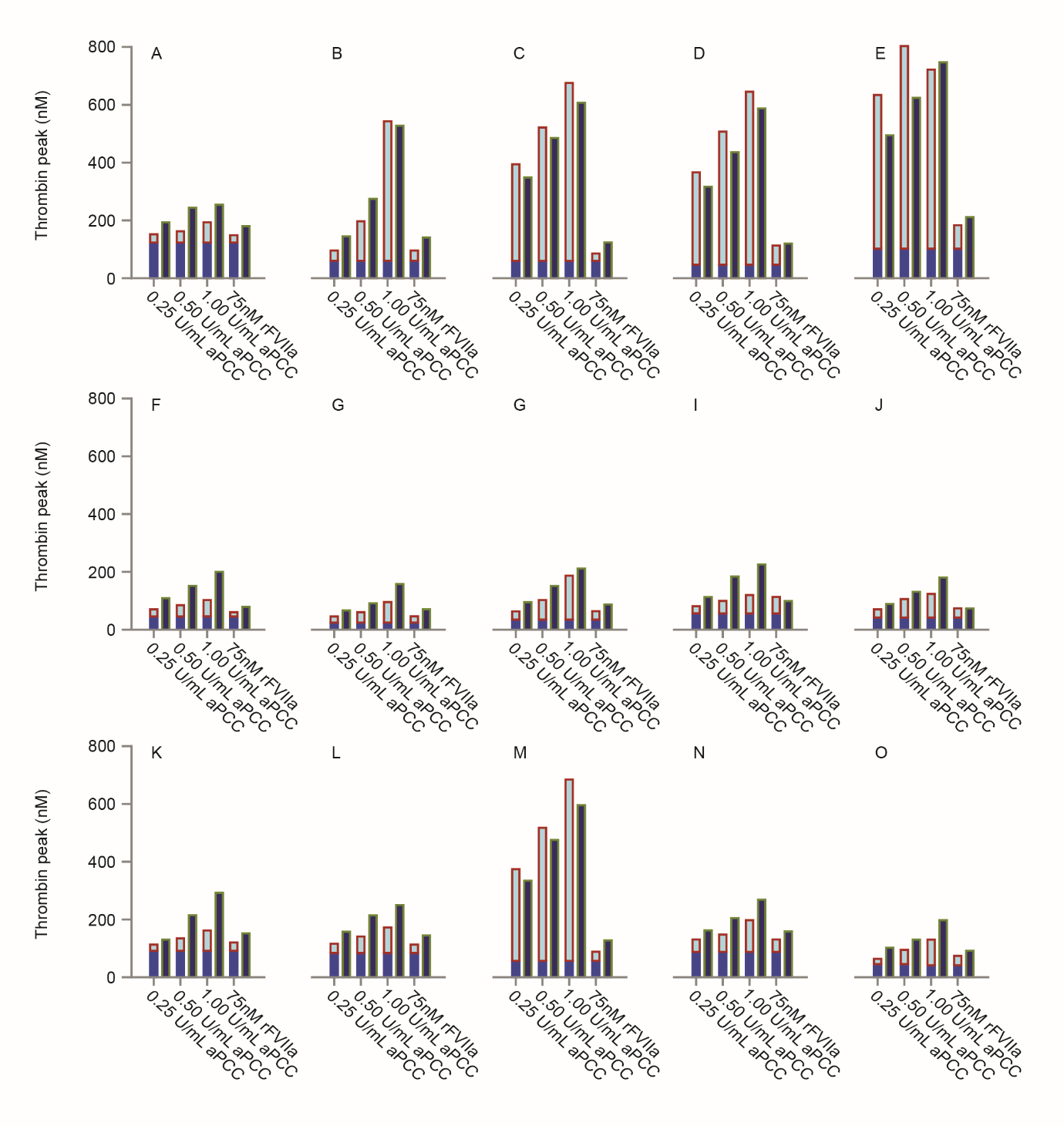


Activated prothrombin complex concentrate (aPCC) or activated recombinant factor VII (rFVIIa) was added to plasma samples from 15 patients with hemophilia A with inhibitors (explorer4 trial, A-O) before concizumab administration and at steady state concizumab prophylaxis. The magnitude of additive effects of concizumab combined with either aPCC or rFVIIa (left columns for each treatment in each patient) were established as the sum of the isolated effect of concizumab at steady-state (lower part of left columns in each group, blue) and the isolated effect of aPCC or rFVIIa when added to pre-dose plasma (upper part of left columns in each group, grey). The total effects observed in samples containing both concizumab and aPCC or rFVIIa are represented by the right column in each group (black). The effect caused by drug-drug interactions was established by comparing the additive effect (left column in each group) with the total observed effect (right column in each group).

**Supplementary Figure 7.** Examples of thrombin peaks after spiking plasma samples from three patients with HA and inhibitors (explorer4 trial) with aPCC.


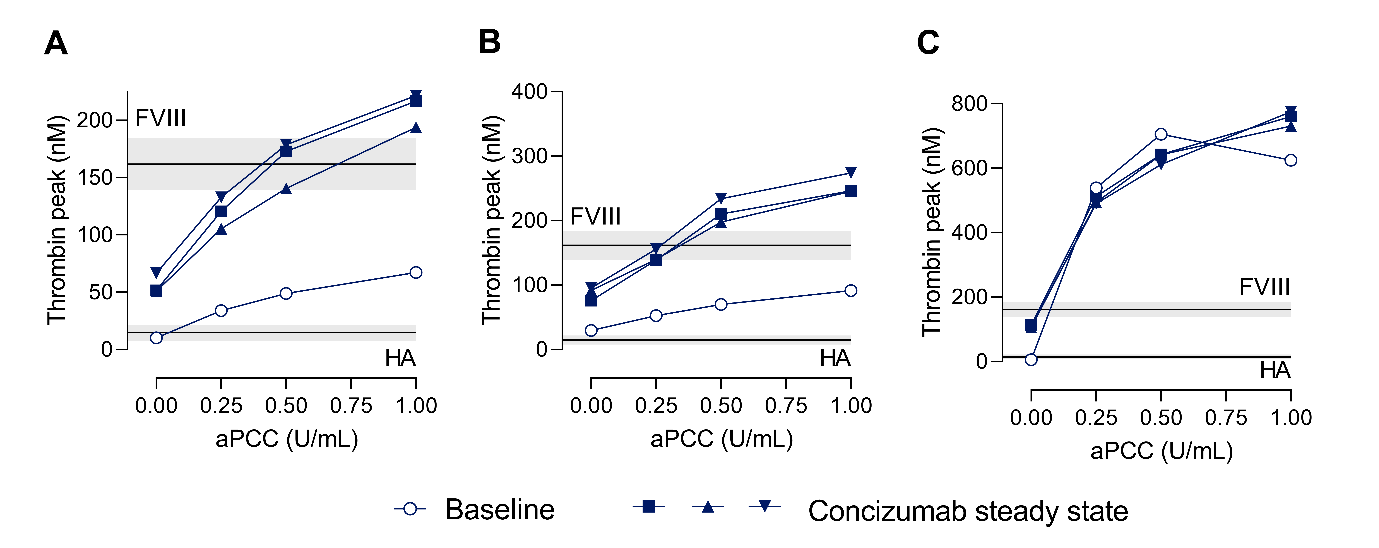


Activated prothrombin complex concentrate (aPCC) 0.25, 0.5 and 1 U/mL was added to baseline plasma samples before concizumab administration and plasma samples obtained at steady state (visits 5, 6, and 7 after 8, 12 and 16 weeks of daily concizumab administration, respectively), and thrombin generation was measured after initiating the assay with 1 pM tissue factor (TF). The area marked ‘FVIII’ represents mean and 95% confidence interval (CI) of thrombin peak after addition of 1 IU/mL rFVIII to non-inhibitor plasma (from the explorer5 trial). The area marked ‘HA’ corresponds to mean and 95% CI of thrombin peak in baseline samples in patients with hemophilia A (HA) and inhibitors (explorer4).

**Supplementary Figure 8.** The magnitude of additive effects on thrombin peak relative to the total effects observed for concizumab and rFVIII in hemophilia A pooled plasma**.**


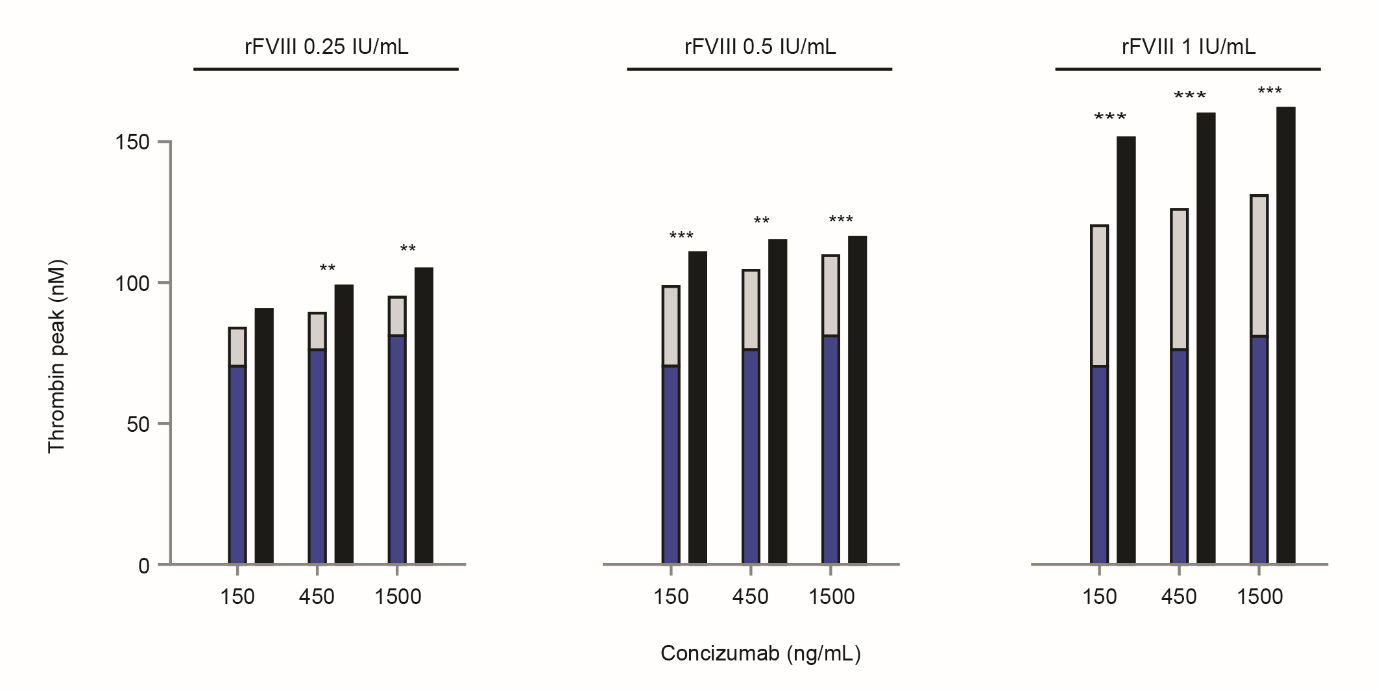


The blue part of the columns on the left-hand side in each pair represents the isolated effect of concizumab, while the upper, grey part within the same columns represents the isolated effect of recombinant factor VIII (rFVIII). Overall, each left column (blue and grey parts together) represents the observed additive effect. The black columns on the right-hand side reflect the effect observed when concizumab and rFVIII were combined. Therefore, the effect caused by drug-drug interaction corresponds to the difference in height of the left and right columns within each pair. Data are mean of 4 experiments. Most combinations of rFVIII and concizumab had a significantly greater effect than the sum of effects of rFVIII and concizumab as indicated with ** (p<0.01) or *** (p<0.001).

**Supplementary Figure 9.** Magnitude of additive effects on thrombin peak relative to the total observed effects of concizumab and rFIX in hemophilia B pooled plasma.


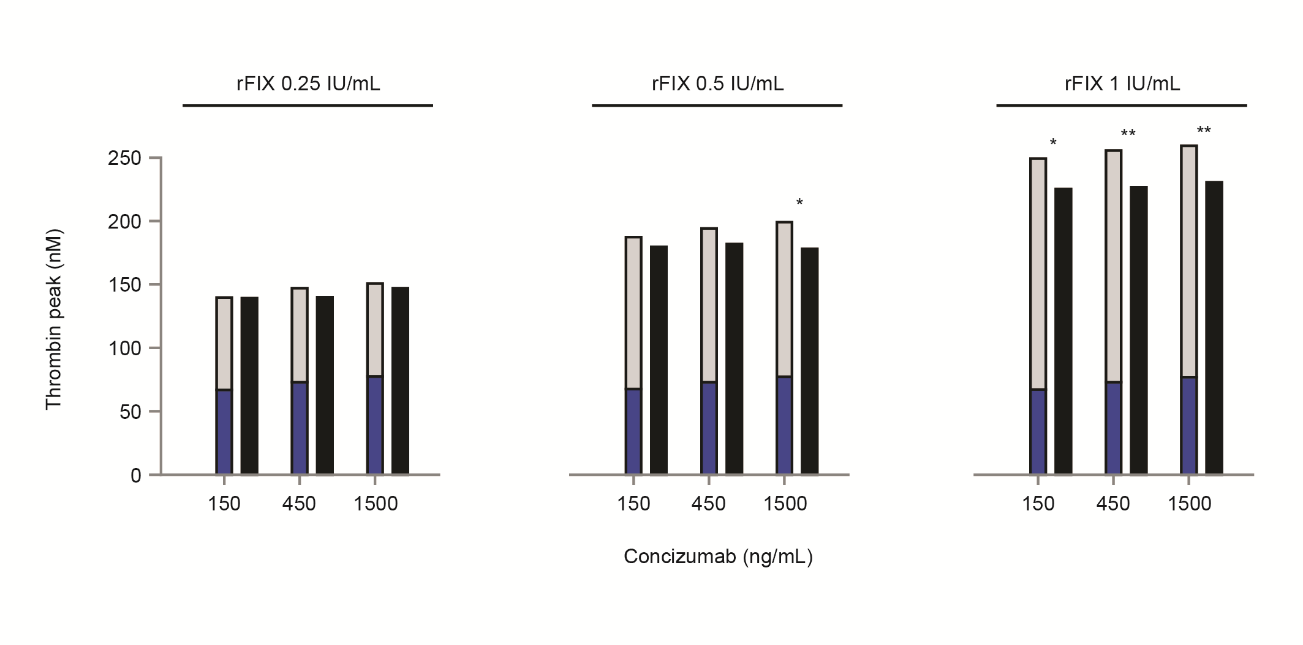


The total observed effects in samples containing both concizumab and recombinant factor IX (rFIX) corresponds to the right columns (black) in each group, while the added isolated effects of concizumab and rFIX correspond to left columns in each group. Within these, the isolated effect of concizumab corresponds to the lower blue part of the columns and the isolated effect of rFIX corresponds to the upper grey part of the columns. Data are mean of 3 experiments. Lower thrombin peak for the observed combined effect of concizumab and rFIX than added isolated effect reflects negative drug-drug interaction, and was significant when marked with * (p<0.05) or ** (p<0.01).
